# Intravenous Delivery of Targeted Liposomes to Amyloid-β Pathology in APP/PSEN1 Transgenic Mice

**DOI:** 10.1371/journal.pone.0048515

**Published:** 2012-10-31

**Authors:** Eric A. Tanifum, Indrani Dasgupta, Mayank Srivastava, Rohan C. Bhavane, Li Sun, John Berridge, Hoda Pourgarzham, Rashmi Kamath, Gabriela Espinosa, Stephen C. Cook, Jason L. Eriksen, Ananth Annapragada

**Affiliations:** 1 Pediatric Radiology, Texas Children’s Hospital, Houston, Texas, United States of America; 2 School of Biomedical Informatics, The University of Texas Health Science Center at Houston, Houston, Texas, United States of America; 3 Department of Pharmacology and Pharmaceutical Sciences, University of Houston, Houston, Texas, United States of America; 4 Department of Radiology, Baylor College of Medicine, Houston, Texas, United States of America; 5 Fieldstone Partners, Houston, Texas, United States of America; University of Manchester, United Kingdom

## Abstract

Extracellular amyloid-β (Aβ) plaques and intracellular neurofibrillary tangles constitute the major neuropathological hallmarks of Alzheimer’s disease (AD). It is now apparent that parenchymal Aβ plaque deposition precedes behavioral signs of disease by several years. The development of agents that can target these plaques may be useful as diagnostic or therapeutic tools. In this study, we synthesized an Aβ-targeted lipid conjugate, incorporated it in stealth liposomal nanoparticles and tested their ability to bind amyloid plaque deposits in an AD mouse model. The results show that the particles maintain binding profiles to synthetic Aβ aggregates comparable to the free ligand, and selectively bind Aβ plaque deposits in brain tissue sections of an AD mouse model (APP/PSEN1 transgenic mice) with high efficiency. When administered intravenously, these long circulating nanoparticles appear to cross the blood-brain barrier and bind to Aβ plaque deposits, labeling parenchymal amyloid deposits and vascular amyloid characteristic of cerebral amyloid angiopathy.

## Introduction

Alzheimer’s disease (AD) is the most common form of dementia in people over the age of 65 and the sixth leading cause of death in the United States. Over 5.4 million Americans suffer from the disease with many more at risk. In 2012, costs associated with care of AD patients were estimated at US$200 billion, not including contributions from unpaid caregivers valued at over US$210 billion [1]. Globally, an estimated 35.6 million people suffer from the disease with an estimated economic impact of US$604 billion in 2010. The global prevalence is projected to increase to 115 million patients by 2050 [2].The diagnosis of probable AD in living patients currently employs a battery of neuropsychological tests, the most common of which is the *mini mental state exam* (MMSE) [3],and a definitive diagnosis requires a post-mortem assessment. The best treatment options currently available only address the symptoms, and not the underlying disease.

Extracellular amyloid-β (Aβ) plaques and intracellular neurofibrillary tangles constitute the major neuropathological hallmarks of AD. Current empirical data suggest that Aβ deposition is associated with the earliest stages of AD [4], [5] and may peak 10 to 20 years prior to Mild Cognitive Impairment (MCI) [6], the earliest clinical manifestation of the disease. There is a significant interest in being able to image potential biomarkers in living patients as a method for both predicting Alzheimer’s disease risk and monitoring progression [7]. Methods to quantify Aβ burden by positron emission tomography (PET) using^18^F and ^11^C-based radiolabeled ligands have been actively developed in recent years [8], with the ^18^F based stilbene (Amyvid) [9] recently receiving FDA approval for use in patients. Although this technique has high sensitivity, PET imaging has significant drawbacks that limit reliability of results, easy access, and availability to patients. For instance, PET agents have relatively coarse spatial resolution with voxels a few millimeters in size [10]. Cerebral amyloid angiopathy (CAA) and the Aβ lesions observed in AD transgenic mice, normal aged humans, and AD patients are microns in diameter, morphologically distinct (mostly diffuse, neuritic, and compact plaques) [11], [12], [13], and not all relevant to definite AD diagnosis [14]. However, all are capable of binding and generating a positive signal from the current PET agents [15], [16], [17]. Preparation of PET agents requires radiochemistry, an expensive process. The radionuclides currently acceptable for medical use have short half-lives (^18^F ∼110 min, ^11^C ∼20 min), limiting imaging time in patients, and the imaging centers that can access the probes. Consequently, advances in diagnostic technology that may improve the quality, resolution, and accessibility of agents that can quantify Aβ in living patients are highly desirable.

In normal individuals, macromolecules and nanoparticles do not pass from the blood to the brain parenchyma. The tightly packed endothelial cell lining of the cerebral vasculature, called the blood-brain barrier (BBB) is responsible for this exclusion, and is also the root cause of low drug delivery efficiency to the brain via the vascular route. In AD, however, there are several pathophysiological changes associated with Aβ deposition that are strongly suggestive of a compromised BBB. These include microvascular poration, a reduction in microvascular density, an increased number of fragmented vessels, and endothelial wall degeneration [18], [19], [20], [21]. Proteins normally sequestered within the blood pool have been reported in the parenchyma. Immunohistochemistry studies in both humans and AD mouse models show elevated levels of plasma-derived proteins such as prothrombin and albumin in vessel walls and in association with parenchymal plaques [22]. In addition to these functional observations of a compromised BBB, there have been direct observations of BBB disruption: Meyer and coworkers demonstrated using Scanning Electron Microscopy, the presence of truncated microvessels and holes with sizes ranging between 0.03 to 3.5 mm^2^ in the dense cortical vasculature in aged APP23 transgenic mice along with significant Aβ deposits [23]. The accumulation of plaques around vessel walls can result in local inflammatory responses, further weakening the integrity of the neurovasculature [21], [24], [25]. In a recent study, Biron and coworkers [26] showed that amyloidogenesis may promote extensive neoangiogenesis, leading to increased vascular permeability and subsequent hypervascularization in brain tissue samples from both a transgenic AD mouse model and human patients.

In light of the pathophysiological and vascular changes associated with AD, we hypothesized that long-circulating nanocarriers such as stealth liposomes could passively extravasate to the abluminal side of the neurovasculature by a mechanism similar to the enhanced permeation and retention effect observed in tumors and inflamed tissue [27]. Following extravasation, the particles should subsequently enter the perivascular drainage pathway by which interstitial fluid and solutes are cleared from the brain [28], [29], [30], [31], [32], and the seeding site for Aβ plaque deposition in CAA [33], [34], [35].

**Figure 1:**
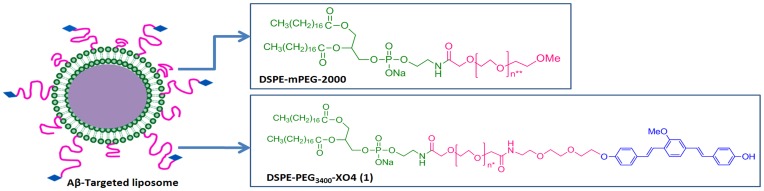
Aβ-Targeted stealth liposome. DSPE-mPEG-2000 lipid conjugate provides stealth properties by preventing facile opsonization and clearance of the particle. DSPE-PEG3400-X04 conjugate imparts Aβ-targeting properties. *Average number of ethylene glycol units = 75. **Average number of ethylene glycol units = 45.

We have previously demonstrated the use of contrast agents encapsulated in liposomes for both computed tomography (CT) [36], and magnetic resonance imaging (MRI) [37], with resolution ∼50 microns. Signal strength is also enhanced in the process: the liposomes encapsulate ∼10^6^ contrast agent molecules, and each liposome can bind one molecular target, thus offering a 10^6^ signal enhancement factor over a single molecule. However, this is dependent on the ability of the liposomes to reach the target. In this study, we therefore tested whether liposomes targeted to amyloid plaques would be able to reach, and bind to, the plaques following i.v. injection. We prepared an Aβ-targeting fluorescent lipid conjugate, 1,2-distearoyl-*sn*-glycero-3-phosphoethanolamine-N-[methoxy-XO4-(polyethylene glycol-3400)] sodium salt (DSPE-PEG_3400_-XO4) (**1**) and incorporated it into stealth liposomes (Figure **1**) in which the highly specific Aβ plaque ligand, methoxy-XO4 (XO4) [38], serves the dual role of the targeting moiety and the fluorescent marker. First, we tested the ability of the particles to bind synthetic Aβ fibrils and their selectivity to bind plaque deposits in AD transgenic mouse brain tissue sections. We then administered them intravenously via tail vein injections to 7- and 12- month old APP/PSEN1 transgenic mice. Animals were sacrificed 72 h post injection and their brains sectioned and analyzed by confocal microscopy. Aβ-specific antibodies were used to identify plaques and confirm the localization of the particles on plaque deposits. To confirm that the liposomes remained intact upon extravasation, and were trafficked intact to the amyloid plaques, we encapsulated Rhodamine in the liposomes, and tested its co-localization with the XO4.

## Results

### Synthesis and Characterization of DSPE-PEG_3400_-XO4 Conjugate

**Figure 2:**
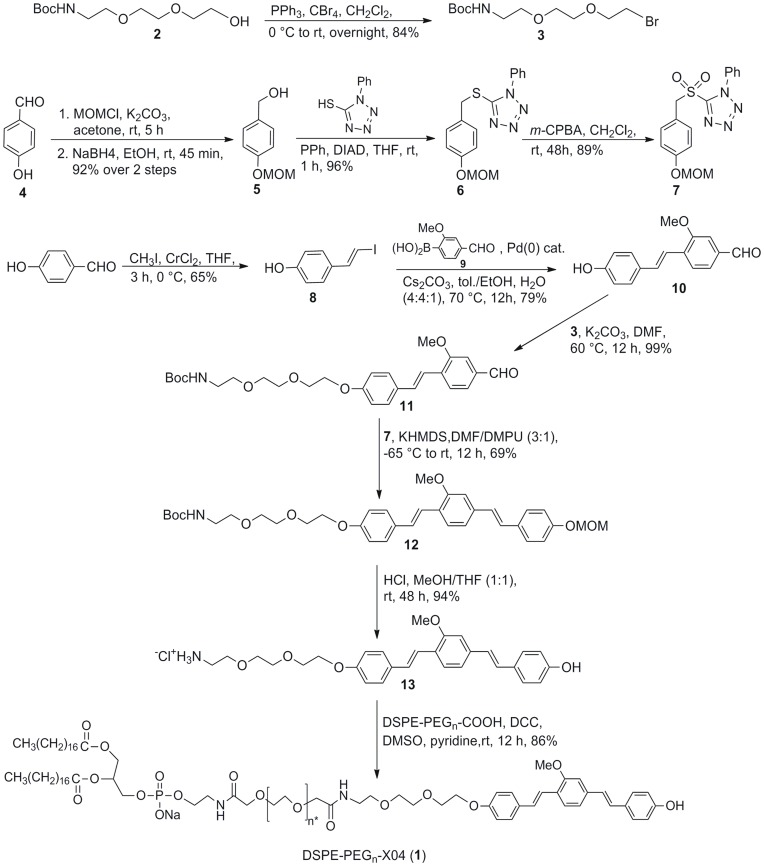
Synthesis route to DSPE-PEG-XO4 conjugate (1). * Average number of ethylene glycol units is 75.

The DSPE-PEG_3400_-XO4 conjugate (**1**), was synthesized as shown in the Figure **2** **.** The synthesis of compound **13**, the linker-X04 moiety, was achieved via a series of Takai [39], Suzuki [40], and Julia-Kocienski olefination [41] reactions. The Boc-protected 3-unit PEG linker precursor bromide (**3**), was prepared from the corresponding commercially available alcohol (**2**). Intermediate **7**, the sulfone for the Julia-Kocienski olefination step was also prepared from 4-hydroxybenzaldehyde as shown. This same starting material was subjected to the standard Takai protocol to yield the corresponding vinyl iodide (**8**). Reaction of **8** with **9** (a commercially available boronic acid) under Suzuki conditions afforded compound **10**. At this point, the linker moiety was attached quantitatively to form aldehyde **11**, which upon exposure to sulfone **7** under optimized Julia-Kocienski conditions gave the desired E,E-alkene (**12**), in 69% yield after column chromatography purification. Global deprotection of the MOM and Boc groups with HCl gave the linker-XO4 moiety (**13**), as the hydrochloride salt. Conjugation to the lipid-PEG moiety proceeded by reacting **13** and DSPE-PEG_3400_-COOH under carbodiimide conditions to afford **1**. All products were characterized by ^1^H and ^13^C NMR, and MALDI mass spectrometry as described in the Methods section.

### Preparation and Characterization of Targeted Liposomes

The synthesis of the fluorescent lipid conjugate DSPE-PEG_3400_-XO4 (**1**), allowed for passive incorporation of the targeting ligand on the surface of the liposomes. A lipid mixture (50 mM) consisting of 1,2-dipalmitoyl-sn-glycero-3-phosphocholine (DPPC), cholesterol, 1,2-distearoyl-sn-glycero-3-phosphoethanolamine-N-[methoxy (polyethylene glycol)-2000] (DSPE-mPEG-2000), and the conjugate (**1**), in a 56.5∶40.0∶3.0∶0.5 molar ratio respectively was dissolved in ethanol. This mixture was hydrated in a histidine (10 mM)/saline (150 mM) buffer (pH 7.5) at 65°C to obtain large, multilamellar vesicles, which were down-sized by sequential extrusion, at 65°C. This approach results in approximately 1∶1 distribution of the conjugate **1**, between the outer and inner bilayers. Dynamic light scattering (DLS), the traditional method for liposomal particle size determination was ineffective because the strong fluorescence of the XO4 interfered with the scattering measurement. Negative stain transmission electron microscopy (TEM) showed a mean diameter of about 150 nm (see supporting information) and,liposomes prepared under identical conditions without the XO4 ligand exhibited a DLS-based particle size consistent with the TEM measurement. Inductively Coupled Plasma-Atomic Emission Spectrometry (ICP-AES) analysis of the phosphorus content indicated a final lipid concentration of 35.5 mM. At this lipid concentration and particle size, an estimated 500 targeting ligands are expected on the surface of each individual liposome. XO4 targeted liposomes encapsulating Rhodamine were prepared by identical methods, except the hydration mixture included 10 mM Rhodamine, and the preparation was conducted under reduced light levels. The stability of each preparation in plasma was assessed as described in the Supplemental Information section (**S18**), and showed no significant leak.

### Binding of Targeted Liposomes to Synthetic Aβ Fibrils

**Figure 3:**
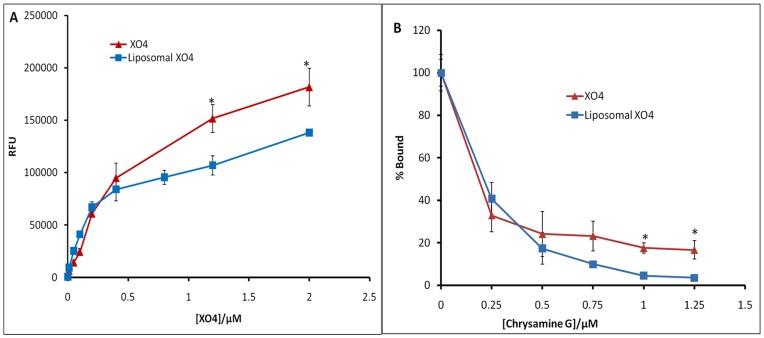
Binding of targeted liposomes to synthetic Aβ(1–40) fibrils. The binding assays were performed in solution by incubating a fixed amount of Aβ fibrils (20 µM) with varying amounts of either Liposome-XO4 or free XO4. The competition experiments were performed by incubating a fixed amount of Aβ fibrils (20 µM) and either Liposome-XO4 or free XO4 (1.0 µM) with varying amounts of Chrysamine G (0 to 1.25 µM). Liposome-XO4-Aβ fibril or XO4-Aβ fibril complexes were then recovered by centrifugation and the bound XO4 measured by fluorometry. **A**: Direct binding of liposome-XO4- or free XO4 to Aβ fibrils. **B**: Competition of Chrysamine G with liposome-XO4 or free XO4 for binding Aβ fibrils. The data shown are means ± S.D. of three independent experiments. The statistical analysis was done by Student’s t test and *p<0.05 was considered significant.

Data demonstrating the *in vitro* binding of targeted liposomes to Aβ aggregates are presented in Figure **3**. The incubation of a fixed concentration of synthetic Aβ_(1–40)_ fibrils (20 µM) with increasing concentrations of liposome-XO4 suspensions (effective XO4 concentration from 0 to 2 µM) or free XO4 showed a corresponding increase in binding, saturating above 0.25 µM for the targeted liposomes and 0.4 µM for the free XO4 (Figure **3A**). These results demonstrate that the coupling of XO4 to liposomes does not significantly affect its ability to interact with Aβ fibrils. Furthermore, the non-fluorescent Chrysamine-G (CG) effectively competes with both liposomal XO4 as well as free XO4 for binding to the Aβ fibrils (Figure **3B**).

### Binding of Liposomal XO4 to Aβ Deposits in AD Mouse Brain Tissue

**Figure 4:**
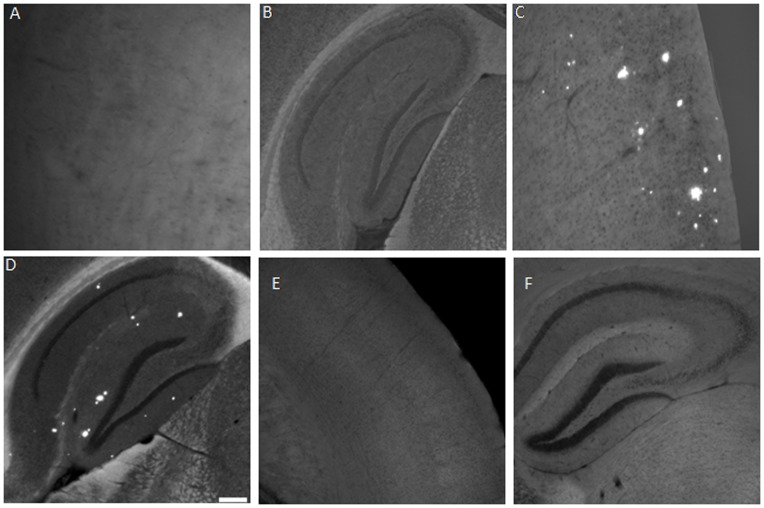
Selective detection of plaque deposits in brain sections of 7 month old APP/PSEN1 mice. Coronal sections (30 µm thick) from non-transgenic and young mice (controls), and 7 month old APP/PSEN1 transgenic mice were incubated in a 3 mM solution of targeted liposomes (effective XO4 concentration = 15 µM) at room temperature, followed by washing with (PBS) to remove unbound liposomes. The stained tissues were then mounted with Vectashield mounting media and viewed under a confocal microscope. Non-transgenic control tissue **A** (cortex) and **B** (hippocampus) show no distinct fluorescence. Dense core plaques are highlighted in the cortex (**C**) and hippocampus (**D**) of treated sections from APP/PSEN1 mice. Scale bar = 100 µm. (**E**) Cortex and (**F**) Hippocampus sections from brains treated with untargeted (i.e. no XO4) liposomes, showing no binding.

Data showing the ability of targeted nanoparticles to selectively bind Aβ plaques in brain tissue sections of APP/PSEN1 mice are shown in Figure **4**. Slides from the non-transgenic control mice (**A, B**) showed no visible fluorescent spots. In the transgenic mice brain sections however, clear, bright fluorescent spots were observed both in the cortex (**C**) and hippocampus (**D**).

**Figure 5:**
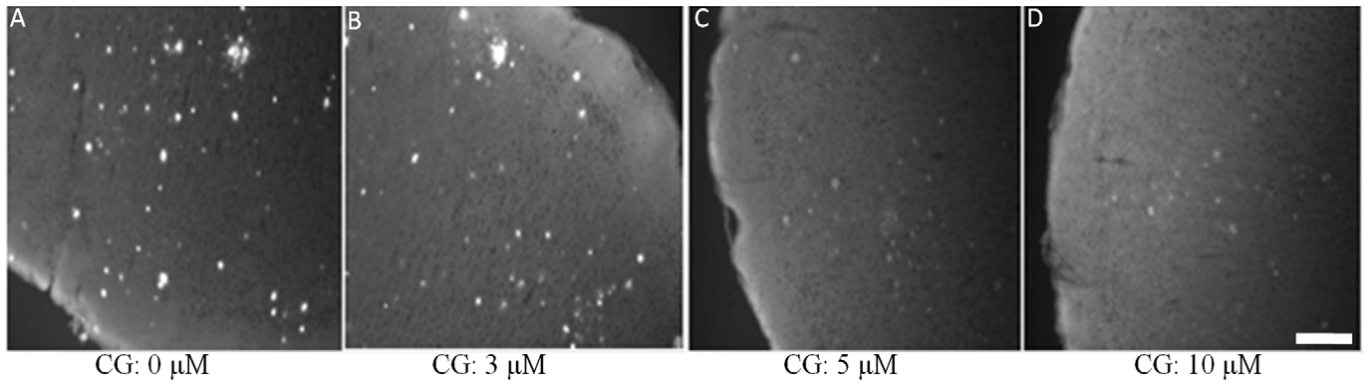
Competition between Chrysamine G (CG) and targeted liposomes for binding sites on Aβ plaques. Brain tissue sections were incubated with 1 µM nanoparticles and increasing concentrations of CG. Scale bar = 100 µm.

The particles were further tested against CG competition to confirm their specificity in binding Aβ plaque deposits in brain tissue. Figure **5** shows the results of this study, which indicate a decrease in fluorescence intensity and the number of labeled plaques with increasing concentration of CG. This parallels the results obtained from competitive binding of the particles and CG to synthetic Aβ fibrils (Figure **3B**), further suggesting that the observed fluorescence on the treated tissues is due to specific binding of particles to Aβ plaque deposits. Taken together with the observed loss of fluorescence from fibrils incubated with the particles and increasing concentrations of CG (Figure **3B**), the data are consistent with the targeted liposomes maintaining affinity and selective binding to Aβ aggregates.

#### Intravenous delivery of targeted liposomes to cortical and hippocampal plaques in APP/PSEN1 transgenic mice

**Figure 6:**
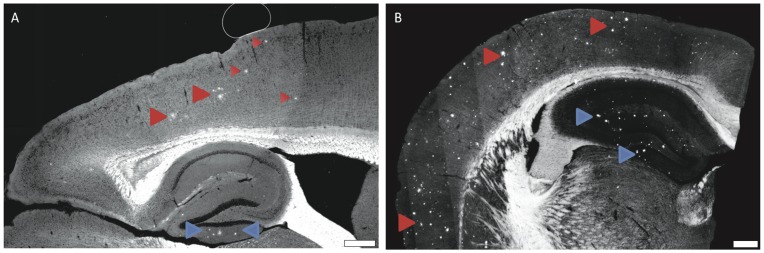
Targeted liposomes label amyloid plaques following intravenous injection. 7 month old mice (**A**) representative of early stage pathology show far fewer plaques than 12 month old mice (**B**) representative of late AD pathology (**B**). Scale bar = 100 µm.

The targeted liposomes were administered to 7 and 12 month old APP/PSEN1 mice by tail vain injection to test the hypothesis that they would cross the BBB, enter the perivascular drainage system, and anchor to Aβ plaque deposits. Seventy-two hours following injection, the mice were sacrificed, and their brains sectioned for fluorescence microscopy. Optically stitched images from these sections show visible amyloid plaques in the hippocampus (blue arrows) and cortex (red arrows) in both the 7 month old (Figure 6A) and the 12 month old (Figure 6B) mice. There is a conspicuous increase in the number of plaques per unit area from the 7 month to the 12 month old mice. No distinct labeling of any kind was observed when non-transgenic mice were injected with the nanoparticles, or when untargeted liposomes were injected as observed in the in vitro experiments.

**Figure 7:**
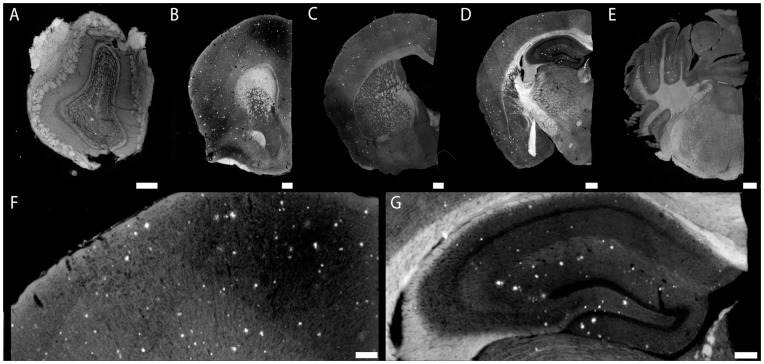
Injected targeted liposomes cross the blood-brain barrier of APP/PSEN1 mice and label parenchymal Aβ deposits. Composite images (**A–E**) of Olfactory bulb (**A**) showing plaque pathology within the granule cell layer. Nanoparticles bound to plaques at the level of the septo-striatum (**B**). Cortical pathology at the septo-diencephalic (**C**). Hippocampal and cortical pathology (**D**) within the caudal diencephalon. Binding to cerebellar plaques (found within APP mice) within the rostral mesencephalon (**E**). An example of cortical plaque pathology (**F**) visualized at 10X magnification. Hippocampal plaque pathology (**G**) is similar to previously reported studies. Scale bar = 100 µm.

**Figure 8:**
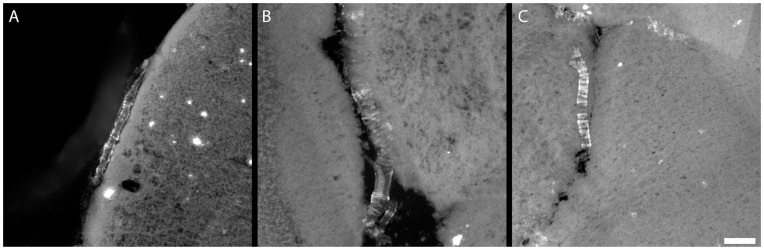
Targeted liposomes bind amyloid deposits in cerebral amyloid angiopathy in 12-month-old APP/PSEN1 mice. Leptomeningial blood vessel (**A**) with prominent amyloid deposits. Prominent CAA (**B**), showing characteristic ring structure, in a large cerebellar blood vessel. Smaller vessels and capillaries (**C**), similarly affected by CAA, are bound by the particles. Scale bar = 100 µm.

Images from 12 month old mice (Figure **7**) reveal that the particles appear to bind amyloid-β deposits across the entire brain. Figure **7A** shows the olfactory bulb, **7B** the septum-striatum, **7C** the septo-diencephalic region, **7D** the caudal diencephalon and **7E** the rostral diencephalon. In each section, plaques bound by nanoparticles are clearly visible as punctate structures suggesting that the targeted nanoparticles label plaque deposits across the entire brain. Vascular amyloid deposits characteristic of CAA are also clearly bound by the agent. Figure **8** shows an example of CAA in 12 month old APP/PSEN1 mice, labeled by the targeted liposomes. This mouse model is known to exhibit clear signs of CAA at this age, particularly in superficial vessels in the *pia mater*.

#### *In vitro* immunofluorescence study of treated mouse brain sections to confirm particle localization on amyloid plaques

**Figure 9:**
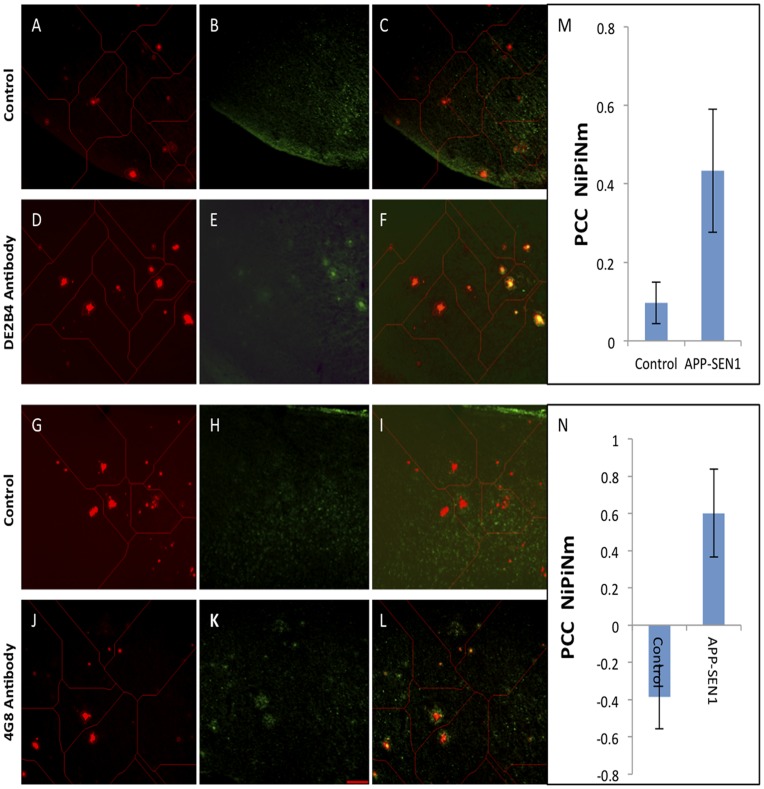
Immunostained brain sections show co-localization of *in* *vivo* targeted liposomes with anti mouse β-amyloid antibodies. Brain sections from mice injected with targeted liposomes (**A, D, G, J**) were probed with two different anti-amyloid antibodies (DE2B4 and 4G8), whose localization was then visualized using a FITC-labeled (**E**) and cy5-tagged Dylight649 (**K**) rabbit-antimouse polyclonal antibody. Control sections (**B** and **H**) were treated with saline instead of the antiamyloid antibody and then identically stained with the respective rabbit-antimouse polyclonal antibodies. Images shown are representatives from 5 slices collected from 4 mice in each group. All images were used for quantitative analysis: In each image, the punctate XO4 signal was used to tessellate the field around each focal plaque (shown by the thin red lines in each image) and create contiguous subdomains within which the colocalization could be assessed. Each subdomain was masked into two regions: the “nuclear” region corresponding to the pixels of the XO4 signal, and the “protein” region, corresponding to all other pixels in the subdomain. Co-localization was then quantified by the Pearson’s Correlation coefficient (PCC) of nuclear intensity versus protein intensity over nuclear mask (PCC NiPiNm). All calculations were performed using Cytseer software (Vala Sciences). The graphs (**M** and **N**) to the right of the images show the PCC values for each treatment. Means and standard deviations calculated over 156 focal plaques for the DE2-B4 antibody and 40 focal plaques for the 4G8 antibody.

*Ex vivo* staining of brain tissue sections from the treated mice using two different anti Aβ antibodies (DE2B4 and 4G8) allowed us to confirm localization of the injected particles against Aβ plaque deposits. The DE2B4 antibody is raised against aa1-17 and 4G8 is raised against aa17-24 of the Aβ protein. As shown in Figure 9, nanoparticle-labeled plaques (D and J) represented by the red signal, show co-localization with DE2B4 (E) and 4G8 (K) in their respective composite images (F and L). Negative controls (B and H) did not show any correlated staining. Graphs M and N show the Pearson’s correlation coefficient (PCC) between the antibody signal and the nanoparticle XO4 signal for each focal plaque.

#### Co-localization of liposome contents with targeting ligand confirming intact trafficking of liposomes to the amyloid plaques

**Figure 10:**
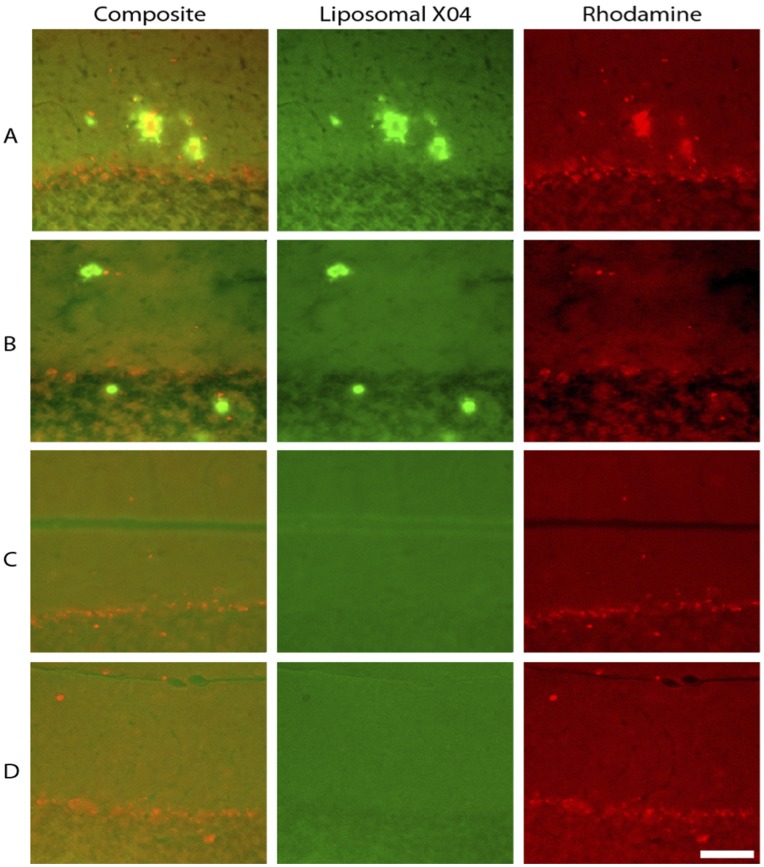
Targeted liposomes bind intact to amyloid plaques when injected *in* *vivo*. To demonstrate that particles bind intact to amyloid plaque pathology, mice were injected with targeted Rhodamine-loaded liposomes, sacrificed 72 h after injection, and the tissue was examined using fluorescence microscopy. **A.** Liposomal X04 and Rhodamine signal co-localize in plaques labeled by Rhodamine-loaded targeted liposomes **B.** Tissue from an APP/PS1 mouse injected with unloaded targeted liposomes shows signal only in the liposomal X04 channel. **C.** tissue from a saline-injected APP mouse, **D.** non-transgenic mouse injected with Rhodamine-loaded XO4 targeted liposomes. Scale bar = 100 µm. Co-localization of the Rhodamine signal (originating with the encapsulated Rhodamine) and the XO4 signal (originating with the XO4 ligand on the surface of the liposome) verifies that the two fluorophores are transported to the sites of amyloid deposition simultaneously, suggesting the liposomes are transported to the site intact.

Rhodamine was encapsulated into XO4-targeted liposomes by the passive loading method, at a concentration of 10 mM. 72 hours after intravenous injection in mice, the animals were sacrificed; the brains fixed and imaged using a DAPI filter set for the XO4 and a TRITC filter set for the Rhodamine. Figure 10 shows representative images from this experiment.

## Discussion

A number of amyloid-beta binding agents have been developed in recent years. Aβ aggregates have multiple binding sites for structurally diverse small molecule ligands [42]. The most prominent of these include Congo red, Chrysamine G (CG), Thioflavin T and their respective derivatives [43]. Some have also been shown to bind to multiple sites on Aβ fibrils albeit with different affinities [44]. Congo red and CG derivatives (including XO4) compete for the same binding pockets while Thioflavin T derivatives compete for separate distinct sites [38], [42], [43]. XO4 was selected as the targeting ligand for this study because of its high affinity and specificity to Aβ aggregates associated with AD. It is symmetrical in shape and its simple stilbene derivatives such as SB-13 [45] maintain high binding affinity to Aβ plaques. This suggested that tethering a linker from either end of the molecule would not adversely affect its binding properties to Aβ aggregates. PEGylation has been shown to have no negative effect on the binding of other Aβ ligands [46]. So the 3-unit PEG linker **3**, was employed to link the ligand to the lipid-PEG moiety. Finally, the intrinsically high fluorescence of the conjugate enabled tracking of the location of particles.

The binding profiles of free XO4 and the particles to synthetic Aβ fibrils (Figure **3A**) appear to be similar. Both show an increase in fluorescence units with a corresponding ligand concentration. However, there is an apparent higher retention of the fluorescent species by fibrils treated with the nanoparticles compared to the corresponding treatment with free XO4. This can be attributed to the fact that each particle bears over 500 XO4 surface ligands, and just one of these may be bound to enable retention of the particle on the fibril. Yet all of these contribute to the observed fluorescence. It is also apparent from the data that liposomal binding appears to saturate at a lower concentration (∼2.5 µM) than the free XO4 (∼4 µM). This is consistent with steric hindrance. XO4 is not expected to suffer from this hindrance at these concentrations due to the small size of the molecule compared to the nanoparticle. These binding properties of the particles versus free XO4 are corroborated by results from the competition experiments with Chrysamine G (Figure **3B**). These show a consistent loss of XO4 fluorescence with increasing concentration of CG albeit with an apparent greater loss by the particles compared to the free XO4. The consistent drop in measured XO4 fluorescence with increasing concentration of CG suggests that both the liposomal XO4 and free XO4 ligands bind to the same site (i.e. the CG site). This further suggests that XO4 binding ability is unchanged by conjugation to the DSPE-PEG anchor. Taken together with the observed loss of fluorescence from plaque-laden brain tissue sections incubated with the particles and increasing concentrations of CG (Figure **5**), these results demonstrate that the targeted liposomes maintain *in vitro* affinity and selective binding to Aβ aggregates.

Confocal microscope images from tissue sections across the entire brains of treated mice (Figure **7**) suggest that the targeted nanoparticles effectively label plaque deposits and CAA (Figure **8**). In co-localization studies, the antibodies stain a wider range around each focal plaque labeled by the particles. This is expected since the antibody labels Aβ peptides while the XO4 only labels aggregates containing prominent beta-sheet structures associated with dense core plaques and CAA. Figure **9** shows the results of counterstaining the brain sections from the treated mice with amyloid antibodies DE2B4 and 4G8. The co-localization of the immunoreactive locations within the images to the XO4 locations is quantified using the Pearson’s Correlation coefficient calculated in tessellated subdomains each containing a focal XO4 signal cluster. When compared to control sections with no amyloid antibody treatment, the significant increase in PCC in the antibody treated specimens confirms the avid co-localization of the two signals. However, the moderate values for the PCC (<0.5) suggest the presence of antibody staining away from the focal XO4 labeled plaques, as well as diffuse tissue background signal in the XO4 channel away from the antibody signal. Examination of the images in Figure **9** suggests the reasons for this. While virtually all the XO4 labeled locations also exhibit antibody signal, there is diffuse antibody staining at locations with no XO4 signal. This is consistent with the affinity of the antibody to oligomeric and even monomeric Aβ, where XO4 as a β-sheet binder is not expected to bind. On the other hand, practically all the immunoreactive dense-core plaques appear to have XO4 signal, suggesting a near universal labeling of focal plaque pathology at the administered lipid dose, but there is diffuse XO4 signal across the tissue, both in the treated and control specimens. The diffuse XO4 signal is therefore attributable to tissue background. The co-localization of XO4 and antibody signal is intriguing, but conceivable, given that, according to the Aβ clearance hypothesis, Aβ accumulates in the AD brain as a result of an imbalance between its production and clearance [47]. Aβ is either cleared from the brain by receptor-mediated transcytosis across the BBB into circulation [48] or via a perivascular pathway. Perivascular clearance has been suggested to be the same pathway by which other macromolecules and solutes are cleared from the brain [28], [29], [30], [31], [32], and represents a mechanism allowing for Aβ plaque seeding and deposition, as observed in CAA [5]. It is possible that once across the BBB, the targeted nanoparticles follow the same clearance route allowing them access to almost all the plaque deposits along this route. Furthermore, Meyer et al [23] have observed that microvasculature in APP23 Tg mice abruptly ended at amyloid plaques, and that plaque deposits sat near or at the end of truncated vessels. These suggest that the plaques would be well accessible to the long circulating nanoparticles, especially if the surrounding tissue is inflamed.

Figure **10** provides verification that the liposomes are actually transported intact, across the BBB and bind to amyloid plaques. By labeling both the liposomal interior compartment (with Rhodamine) and the bilayer membrane (with XO4), and injecting these liposomes i.v. in transgenic mice, co-localization of the two tracers at amyloid plaque sites can be considered an indication of intact transport of the liposomal particles to the plaques. This is demonstrated in Figure **10A**. Figures **10B, 10C and 10D** are the corresponding controls, (**10B**: transgenic mouse treated with XO4 targeted liposomes, with no Rhodamine, **10C**: transgenic mouse treated with saline, **10D**: non-transgenic mouse treated with Rhodamine-XO4 liposomes). Clearly, amyloid plaques are visible in the XO4 images in **10A** and **B**, with co-localization of the Rhodamine signal in **10A** demonstrating that fluorescent Aβ targeted liposomal nanocarriers can cross the BBB and bind to Aβ pathologies in an AD mouse model. Delivery of any exogenous agents to the brain from the vascular compartment is usually hindered by the effectiveness of the BBB in restricting transport to the CNS. That these particles are able to cross the BBB and label amyloid plaques in as young as 7-month old APP/PSEN1 transgenic mice has multiple implications. Significantly, liposomal nanocarriers have previously been shown to be versatile, safe, and effective delivery vehicles for many drugs ranging from small molecules to proteins (both enzymatic and non-enzymatic) [49]. The fact that these particles can be loaded with CT [36] or MR [37] contrast agent payloads suggests the possibility of using either CT or MR for the imaging of amyloid deposits. Since the particles can carry up to 10^6^ molecules of contrast agent each, they can effectively increase signal by up to a factor of 10^6^ over that of conventional CT or MR agents. Coupled with the exceptional spatial resolution of MRI [37], this agent should allow for the visualization of features at a voxel size of 30 µm, providing superior spatial resolution, allowing for the separation of parenchymal plaques from CAA. Clearly, the *contrast* developed in such images will result from a balance between the rapid clearance of unbound species and the relatively slow clearance of bound species from the brain. One anticipates that unbound species would clear by the normal mechanisms of CSF transport, dominated by CSF convection in the arachnoid space, driven by carotid pulsation. The other transport mechanisms such as transcytosis and perivascular clearance probably have similar time constants, but also account for far lower total flux. With binding constants in the micromolar range, dissociation is quite likely to occur after bulk clearance takes place, enabling longitudinal imaging. Similarly, the therapeutic payload capacity of stealth liposomes has been extensively demonstrated [49]. We therefore suggest the potential for the use of these particles as drug carriers to the site of amyloid deposits.

### Conclusions

This study reports the preparation of Aβ-targeted stealth liposomal nanoparticles using the Aβ-targeted lipid conjugate DSPE-PEG-XO4. These nanoparticles maintain similar binding profiles to Aβ_(1–40)_ as the free XO4 ligand *in vitro*. They selectively bind to amyloid deposits in brain tissue sections of APP/PSEN1 transgenic mice *in vitro*. Ex vivo analyses of treated brain tissue show that when injected into these mice, the targeted particles efficiently bind both parenchymal plaques and CAA associated amyloid throughout the brain. *In vitro* immunohistochemistry, and verified co-localization of both the liposome encapsulate and bilayer membrane components performed on brain tissue sections obtained from treated animals confirmed the ability of the particles to traverse the BBB and bind amyloid-β plaque deposits. A fundamental limitation of this work is the fact that the visualization of liposome localization was done ex vivo, after washing of specimens, a process that will necessarily wash away unbound liposomes, and reduce the background signal. When used in vivo, the imaging will not have this luxury, and it remains to be seen whether the bound liposome signal will be discernible above the unbound background. There are currently two reports of liposomal preparations with affinity to Aβ fibrils *in vitro* [50], [51]. The theme of both reports is to develop Aβ aggregate clearance systems, and not as a delivery system to the sites of amyloid plaque. To the best of our knowledge, the results of the present study are the first report of successful intravenous delivery of Aβ-targeted liposomal nanocarriers to both parenchymal plaques and CAA in a preclinical model of Alzheimer’s disease.

## Methods

### Chemical Synthesis

#### General

2-[2-(2-Boc-aminoethoxy)ethoxy]ethanol (**2**) was purchased from CiVentiChem, Inc. and 4-Formyl-2-methoxyphenylboronic acid (**9**) was purchased from AOBChem, Inc. All other reagents were obtained from Sigma-Aldrich or Acros Organics and used without further purification. Proton nuclear magnetic resonances (^1^H NMR) were recorded at 300 MHz or 500 MHz on Bruker 300 or 500 NMR spectrometers. Carbon nuclear magnetic resonances (^13^C NMR) were recorded at 75 MHz or 125 MHz on a Bruker 300 or 500 NMR spectrometers respectively. Chemical shifts are reported in parts per million (ppm) from an internal standard acetone (2.05 ppm), chloroform (7.26 ppm), or dimethylsulfoxide (2.50 ppm) for ^1^H NMR; and from an internal standard of either residual acetone (206.26 ppm), chloroform (77.00 ppm), or dimethylsulfoxide (39.52 ppm) for ^13^C NMR. NMR peak multiplicities are denoted as follows: s (singlet), d (doublet), t (triplet), q (quartet), bs (broad singlet), dd (doublet of doublet), tt (triplet of triplet), ddd (doublet of doublet of doublet), and m (multiplet). Coupling constants (*J*) are given in hertz (Hz). High resolution mass spectra (HRMS) were obtained from The Ohio State University Mass Spectrometry and Proteomics Facility. Thin layer chromatography (TLC) was performed on silica gel 60 F_254_ plates from EMD Chemical Inc. and components were visualized by ultraviolet light (254 nm) and/or phosphomolybdic acid, 20 wt% solution in ethanol. SiliFlash silica gel (230–400 mesh) was used for all column chromatography.

#### 2-[2-(2-bromoethoxy)ethoxy]-N-Boc-ethanamine (3)

To a solution of alcohol **2** (1.00 g, 4.01 mmol) and PPh_3_ (1.37 g, 5.21 mmol) in CH_2_Cl_2_ (20 mL) at 0°C, under positive N_2_ pressure, was added a solution of carbontetrachloride (1.73 g, 5.21 mol) in CH_2_Cl_2_ (10 mL). The mixture was stirred at 0°C for a further 30 min, after which it was allowed to come to room temperature overnight. The solvent was removed *in vacuo* to obtain a white paste. Ethyl acetate (10 mL) was added and the resulting mixture shaken vigorously to obtain a white precipitate. This was filtered off and the ensuing filtrate containing the bromide was concentrated and purified by flash column chromatography on silica gel eluted with 25% ethyl acetate/hexanes to obtain previously reported **3** [52] as colorless oil (841 mg, 84% yield). ^1^H NMR (CDCl_3_, 500 MHz) δ 4.99 (bs, NH), 3.75 (t, *J* = 6.0 Hz, 2H), 3.60 (m, 4H), 3.49 (t, *J* = 5.2 Hz, 2H), 3.42 (t, *J* = 6.3 Hz, 2H), 3.25 (bs, 2H), 1.39 (s, 9H); ^13^C NMR (CDCl_3_, 125 MHz) δ 155.92, 79.12, 71.14, 70.39, 70.22, 70.14, 40.34, 30.20, 28.39; HRMS clcd for C_11_H_22_BrNO_4_Na^+^ *m/z* (M+Na) 334.0630, found 334.0631.

#### [4-(methoxymethoxy)phenyl]methanol (5)

To a stirred solution of 4-hydroxybenzadehyde (5.00 g, 40.93 mmol) in anhydrous acetone (100 mL) at ambient temperature were added anhydrous K_2_CO_3_ (11.32 g, 81.87 mmol), followed by MOMCl (3.4 mL, 45.04 mmol). The resulting mixture was stirred for 5 h followed by filtration to remove inorganic salts. Acetone was then stripped from the filtrate on a rotary evaporator and the resulting crude product dissolved in ethyl acetate, washed with saturated NH_4_Cl solution and brine, and dried over Na_2_SO_4_. Removal of the solvent by rotary evaporation gave the desired MOM-protected 4-hydroxybenzaldehyde (6.82 g, 100% yield) which was used in the next step without further purification.

MOM-protected 4-hydroxybenzaldehyde (3.00 g, 18.06 mmol) was dissolved in ethanol (30.0 mL). To this was added NaBH_4_ (704.8 mg, 18.63 mmol) in one portion. The resulting mixture was stirred at ambient temperature for 45 min at which point it was cooled in an ice bath and excess acetone added drop wise to quench unreacted NaBH_4_. The solvents were then removed by rotary evaporation and the residue dissolved in ethyl acetate, rinsed with saturated NH_4_Cl solution and brine, and dried over Na_2_SO_4_. Removal of solvent followed by flash column chromatography on silica gel eluted with a mixture of ethyl acetate/hexanes (3∶7) gave the previously reported MOM-protected 4-hydroxybenzyl alcohol, **5** (2.80 g, 92% yield). ^1^H NMR (CDCl_3_, 300 MHz) δ 7.28 (d, *J* = 8.7 Hz, 2H), 7.03 (d, *J* = 8.7 Hz, 2H), 5.17(s, 2H), 4.59(s, 2H), 3.48 (s, 3H).

#### 5-[4-(methoxymethoxy)benzylthio]-1-phenyl-1H-tetrazole (6)

To a stirred mixture of alcohol **5** (5.77 g, 34.31 mmol), PPh_3_ (9.90 g, 37.74 mmol), and 1-phenyl-H-tetrazole-5-thiol (7.34 g, 41.17 mmol), in THF (200 mL) at was added DIAD (8.6 mL, 41.17 mmol) drop wise. The mixture was stirred for 45 min, after which the reaction was judged complete by TLC. The reaction was then quenched with saturated NH_4_Cl solution, extracted with ether, rinsed with brine, and dried over anhydrous Na_2_SO_4_. Following filtration, the solvent was removed *in vacuo* to give a crude mixture which was purified by chromatography on silica gel eluted with 20% ethylacetate/hexanes to obtain **6** (10.80 g, 96% yield) as a white solid. ^1^H NMR (CDCl_3_, 500 MHz) δ 7.52 (m, 5H), 7.34 (d, *J* = 8.5 Hz, 2H), 6.98 (d, *J* = 8.5 Hz, 2H), 5.15 (s, 2H), 4.58 (s, 2H), 3.45 (s, 3H); ^13^C NMR (CDCl_3_, 125 MHz) δ 157.25, 154.08, 133.77, 130.64, 130.19, 129.86, 128.50, 123.91, 116.65, 94.46, 56.13, 37.35; HRMS clcd for C_16_H_16_N_4_O_2_SNa^+^ *m/z* (M+Na) 351.0892, found 351.0879.

#### 5-[4-(methoxymethoxy)benzylsulfonyl]-1-phenyl-1H-tetrazole (7)

To a solution of sulfide **6** (10.50 g, 31.98 mmol), in dichloromethane (300 mL) at 0°C was added *m*-CPBA (70–75% in water, 39.40 g), and the mixture allowed to stir at room temperature for 48 h. 10% sodium thiosulfate solution (100 mL) was added and the resulting mixture stirred for 30 min. The two phases were separated and the organic phase washed with saturated NaHCO_3_, rinsed with brine, dried over Na_2_SO_4_ and concentrated to obtain a white solid. This was further purified on a silica gel column eluted with 40% ethyl acetate/hexanes to obtain sulfone **7** (10.10 g, 89% yield).^ 1^H NMR (CDCl_3_, 500 MHz) δ 7.58 (t, *J* = 7.5 Hz, 1H), 7.51(t, *J* = 7.5 Hz, 2H), 7.36 (d, *J* = 7.5 Hz, 1H), 7.25 (d, *J* = 8.5 Hz, 2H), 7.02 (d, *J* = 8.5 Hz, 2H), 5.19 (s, 2H), 4.89 (s, 2H), 3.48 (s, 3H); ^13^C NMR (CDCl_3_, 125 MHz) δ 158.53, 153.06, 133.02, 132.94, 131.43, 129.45, 125.39, 117.68, 116.86, 94.30, 61.88, 56.21. HRMS clcd for C_16_H_16_N_4_O_4_SNa^+^ *m/z* (M+Na) 383.0790, found 383.0785.

#### (E)-4-(2-iodovinyl)phenol (8)

CrCl_2_ (12.08 g, 98.26 mmol) was placed in a nitrogen-flushed dry flask and flame-dried under high vacuum. After cooling to room temperature, THF (90 mL) was added and the resulting suspension cooled to 0°C under positive N_2_. A mixture of iodoform (19.34 g, 49.13 mmol) and 4-hydroxybenzaldehyde (3.00 g, 24.56 mmol) in THF (50 mL) was added and the resulting mixture stirred at 0°C for 3 h. It was then poured into brine (100 mL) and the two phases separated. The aqueous phase was extracted with CH_2_Cl_2_ (3×50 mL) and the combined organic extracts dried (Na_2_SO_4_), concentrated and chromatographed on silica gel eluted with a mixture of ethyl acetate/hexanes (1∶9). Fractions containing the product were concentrated to obtain a white solid which was recrystallized in hexanes to yield previously reported iodide (**8**) [53] as golden flakes (5.31 g, 65% yield, 93∶7 E/Z). ^1^H NMR (CDCl_3_, 300 MHz) δ 7.24 (d, *J* = 15.0 Hz, 1H), 7.08 (d, *J* = 8.7 Hz, 2H), 6.75 (d, *J* = 8.7 Hz, 2H), 6.49 (d, *J* = 15.0 Hz, 1H); ^13^C NMR (CDCl_3_, 75 MHz), δ 157.11, 144.43, 141.95, 129.86, 127.30, 115.57.

#### (E)-4-(4-hydroxystyryl)-3-methoxybenzaldehyde (10)

A mixture vinyl of iodide **8** (200.0 mg, 0.81 mmol), 4-formyl-2-methoxyphenyl boronic acid (176.0 mg, 0.98 mmol), Cs_2_CO_3_ (794 mg, 2.44 mmol), and [1,1'-Bis(diphenylphosphino)-ferrocene]dichloropalladium (II) complexed with dichloromethane (19.9 mg, 0.02 mmol) in toluene/ethanol/water (4∶4:1, 9 mL) was deoxygenated by bubbling N_2_ through it for 30 min. It was then heated at 70°C overnight, after which TLC showed complete consumption of the iodide. Upon cooling to ambient temperature, it was diluted with water (5 mL) and extracted with ethylacetate, dried over Na_2_SO_4_, concentrated, and chromatographed on silica gel column eluted with ethylacetate/hexanes (2∶8) to obtain **10** as a pale yellow solid (163.0 mg, 79% yield). ^1^H NMR (DMSO-d_6_, 300 MHz) δ 9.95 (s, 1H), 9.71 (s, 1H), 7.84 (d, *J* = 7.2 Hz, 1H), 7.47–7.55 (m, 4H), 7.35 (d, *J* = 16.6 Hz, 1H), 7.24 (d, *J* = 16.6 Hz, 1H), 6.80 (d, *J* = 8.4 Hz, 2H), 3.93 (s, 3H); ^13^C NMR (DMSO-d_6_, 75 MHz), δ 192.17, 157.94, 156.40, 135.68, 132.49, 132.40, 128.36, 128.02, 126.06, 123.15, 118.33, 115.72, 110.46, 55.72; HRMS clcd for C_16_H_14_O_3_Na^+^ *m/z* (M+Na) 277.0841, found 277.0850.

#### (E)-4-{4-[2-(2-(2-N-Boc-aminoethoxy)ethoxy)ethoxy]styryl}-3-methoxybenzaldehyde (11)

A mixture of compounds **10** (733 mg, 2.88 mmol), **3** (1.08 g, 3.46 mmol), K_2_CO_3_ (1.20 g, 8.65 mmol), and anhydrous DMF (20 mL) were placed in a boiling tube, sealed and heated at 70°C for 5 h. Upon cooling to room temperature, the white solid was filtered off. The filtrate was diluted with ethyl acetate, washed (saturated NH_4_Cl solution and brine), dried (Na_2_SO_4_), concentrated and chromatographed on silica gel eluted with 40% ethyl acetate/hexanes to yield **11** (1.39 g, 99% yield). ^1^H NMR (CDCl_3_, 500 MHz) δ 9.97 (s, 1H), 7.75 (d, *J* = 8.0 Hz, 1H), 7.52 (d, *J* = 8.5 Hz, 2H), 7.47 (d, *J* = 8.0 Hz, 1H ), 7.42 (s, 1H), 7.39 (d, *J* = 16.5 Hz, 1H), 7.23 (d, *J* = 16.5 Hz, 1H), 6.95 (d, *J* = 8.5 Hz, 2H), 4.20 (t, *J* = 4.5 Hz, 2H), 3.98 (s, 3H), 3.90 (t, *J* = 4.5 Hz, 2H), 3.75 (t, *J* = 4.5 Hz, 2H), 3.68 (m, 2H), 3.58 (t, *J* = 5.0 Hz, 2H), 3.35 (bs, 2H), 1.46 (s, 9H); ^13^C NMR (CDCl_3_, 125 MHz) δ 191.74, 159.10, 157.19, 136.17, 133.56, 132.14, 130.43, 128.98, 126.25, 124.53, 120.38, 112.03, 109.49, 70.94, 70.49, 69.87, 67.63, 55.87, 40.53, 28.56; HRMS clcd for C_27_H_35_NO_7_Na^+^ *m/z* (M+Na) 508.2311, found 508.2312.

#### (E,E)-2-{2-[2-(4-(2-methoxy-4-(4-(methoxymethoxy)styryl)styryl)phenoxy)ethoxy]ethoxy}-N-Boc-ethanamine (12)

A solution of KHMDS (1 M in THF, 7.2 mL, 7.2 mmol) was added slowly to a stirred solution of sulfone **7** (2.06 g, 5.72 mmol) in DMF/DMPU (3∶1, 28 mL), in a flask wrapped with aluminum foil and maintained below −65°C. This was followed immediately by addition of the aldehyde **11** (1.39 g, 2.86 mmol) dissolved in DMF (6 mL). With temperature maintained below −65°C, the mixture was stirred for 3 h, then allowed to warm to ambient temperature overnight. The resulting mixture was poured into saturated NH_4_Cl solution, extracted with ether, rinsed with water and brine, and dried over anhydrous MgSO_4_. Upon filtration of the drying agent, the solvents were removed *in vacuo* to give a crude product which was purified by passing through a silica gel column eluted with 50% ethyl acetate in hexanes as eluent to obtain 1.22 g of the desired *E,E*-isomer **12** (69% yield) as a yellow solid. ^1^H NMR (acetone-d_6_, 500 MHz) δ 7.62 (d, *J* = 8.0 Hz, 1H), 7.54 (d, *J* = 8.5 Hz, 2H), 7.50 (d, *J* = 8.5 Hz, 2H ), 7.37 (d, *J* = 17.5 Hz, 1H), 7.25 (d, *J* = 16.5 Hz, 1H), 7.24 (s, 1H), 7.20 (d, *J* = 16.5 Hz, 1H), 7.17 (d, *J* = 8.0 Hz, 1H), 7.12 (d, *J* = 17.5 Hz, 1H), 7.05 (d, *J* = 8.5 Hz, 2H), 6.96 (d, *J* = 8.5 Hz, 2H), 5.22 (s, 2H), 4.16 (t, *J* = 4.5 Hz, 2H), 3.96 (s, 3H), 3.83 (t, *J* = 5.0 Hz, 2H), 3.67 (t, *J* = 4.0 Hz, 2H), 3.61 (m, 2H), 3.51 (t, *J* = 5.5 Hz, 2H), 3.44 (s, 3H), 3.23 (m, 2H), 1.40 (s, 9H); ^13^C NMR (CDCl_3_, 125 MHz) δ 160.54, 159.01, 158.94, 139.95, 133.12, 132.78, 129.98, 129.79, 129.51, 129.46, 128.63, 127.95, 127.58, 122.69, 121.00, 118.27, 116.62, 110.53, 96.04, 79.56, 72.34, 71.93, 71.65, 71.30, 69.37, 57.03, 56.91, 42.05, 29.60; HRMS clcd for C_36_H_45_NO_8_Na^+^ *m/z* (M+Na) 642.3043, found 642.3051.

#### 4-(4-(4-(2-(2-(2-aminoethoxy)ethoxy)ethoxy)styryl)-3-methoxystyryl)phenol hydrochloride (13)

Compound **12** (550 mg, 0.89 mmol) was dissolved in a mixture of THF/MeOH (1∶1, 120 mL) and cooled to 0°C. To this was added concentrated HCl (6 mL) and the resulting mixture allowed to stir at room temperature for 40 h. Dry K_2_CO_3_ (5.0 g) was added and the mixture stirred for a further 30 min. The solid was filtered off and the filtrate concentrated down to 20 mL, at which point a pale yellow precipitate forms. This was cooled in an ice bath for 15 min and the solid filtered, rinsed with cold acetone, and dried to obtain **13** (428 mg, 94% yield). ^1^H NMR (DMSO-d_6_, 500 MHz) δ 8.10 (s, NH_3_^+^), 7.60 (d, *J* = 8.0 Hz, 1H), 7.49 (d, *J* = 8.5 Hz, 2H), 7.43 (d, *J* = 8.5 Hz, 2H), 7.26 (d, *J* = 16.5 Hz, 1H), 7.23-7.14 (m, 4H), 7.01 (d, *J* = 16.5 Hz, 1H), 6.95 (d, *J* = 8.5 Hz, 2H), 6.80 (d, *J* = 8.5 Hz, 2H), 4.11 (t, *J* = 4.4 Hz, 2H), 3.91 (s, 3H), 3.76 (t, *J* = 4.5 Hz, 2H), 3.60 (m, 6H), 2.95 (m, 2H); ^13^C NMR (CDCl_3_, 125 MHz) δ 158.50, 157.94, 157.07, 138.50, 130.86, 129.00, 128.56, 128.33, 128.08, 126.57, 125.50, 125.12, 120.99, 119.38, 116.12, 115.25, 109.11, 70.26, 70.19, 69.43, 67.65, 67.14, 56.04, 38.34; HRMS clcd for C_29_H_34_NO_5_^+^ *m/z* (M-Cl) 476.2437, found 474.2433.

#### DSPE-PEG3400-XO4 conjugate (1)

DSPE-PEG_3400_-COOH (100 mg, 0.02 mmol) was dissolved in 2 ml anhydrous DMSO, followed by the addition of DCC (14.9 mg, 0.07 mmol) and pyridine (0.5 mL), and the mixture allowed to stir at room temperature for 10 min. Compound **13** (18.5 mg, 0.04 mmol) was then added and the reaction mixture allowed to stir overnight. The pyridine was azeotroped off with 5 mL EtOH on a rotary evaporator maintained at 50°C. The resulting residue was diluted with 10 mL water and centrifuged (3 times) at 1100 rpm for 15 min to remove dicyclohexyl urea by product. It was then dialyzed with 2000 MWCO dialysis cassette twice against 2 l 50 mM saline, then trice against 2 l water. This was then lyophilized to yield 95.8 mg (86% yield) of the conjugate as a pale yellow solid. Its identity and purity was confirmed by ^1^H NMR and MALDI mass spectrometry.

### Preparation of Targeted Liposomes

A lipid mixture consisting of 1,2-dipalmitoyl-sn-glycero-3-phosphocholine (DPPC), cholesterol, 1,2-distearoyl-sn-glycero-3-phosphoethanolamine-N-[methoxy (polyethylene glycol)-2000] (DSPE-mPEG-2000), and DSPE-PEG-3400-XO4 in a molar ratio of 56.5∶40.0∶3.0∶0.5 was dissolved in ethanol (600 µL) and slowly warmed in a water bath to obtain a clear solution. This was then transferred to a histidine/saline buffer, pH 7 (5 mL) at 65°C and allowed to hydrate for 45 min, followed by extrusion on a Lipex thermoline extruder (Northern Lipids Inc., Canada), with five passes through a 200 nm Nuclepore membrane (Waterman, Newton, MA) followed by ten passes through a 100 nm membrane. The resulting preparation was subjected to diafiltration through a 100,000 MWCO membrane to remove any free floating lipids or unencapsulated materials. Mean particle size was determined by transmission electron microscopy.

### Synthesis of Aβ Fibrils

Beta-Amyloid_(1–40)_ peptide was purchased from rPeptide (Bogart, GA). The fibrils were prepared following the protocol outlined by Klunk *et al* [38]. Aβ_(1–40)_ was dissolved in PBS, pH 7.4 to a final concentration of 433 µg/ml (100 µM). The solution was stirred using a magnetic stir bar at 700 rpm for 4 days at room temperature to drive the formation of fibrils. The stock solution was aliquoted and stored at −80°C for future use. The stock solutions were stirred thoroughly before removing aliquots for binding assays, to maintain a homogenous suspension of fibrils.

### Binding Affinity of Targeted Liposomes to Synthetic Aβ Fibrils

Targeted liposomes and XO4 stock solutions were diluted with 10 mM Tris-HCl, pH 7.4 to 500 nM. A small volume of the 100 µM Aβ stock solution was added to the test compounds to achieve a final fibril concentration of 20 µM. This was followed by addition of appropriate concentrations of the non-fluorescent competitor Chrysamine G. The binding mixture was incubated at room temperature for 1 h and then centrifuged for 20 min at 16,400 rpm to separate the fibrils. The precipitate was washed twice with Tris-HCl, and resuspended in the buffer. Fluorescence was measured in a SpectraMax-384 plate reader using excitation and emission wavelengths of 368 nm and 450 nm respectively.

### Animal Studies

All animal studies reported in this paper were conducted under study-specific protocols that were specifically approved by the Institutional Animal Care and Use Committee (IACUC) at the University of Houston. The B6.C6-Tg(APPswe,PSEN1dE9)85Dbo/J (APP/PSEN1; Jackson Laboratories) mouse line was used in this study. These double transgenic mice express a chimeric mouse/human amyloid precursor protein (Mo/HuAPP695swe) and a mutant human presenilin 1 (PS1-dE9) both directed to CNS neurons and associated with early-onset Alzheimer's disease. These mice progressively develop cortical and hippocampal Aβ plaques, accompanied by cerebral amyloid angiopathy, in an age-related manner similar to that observed in human patients [54], [55], [56]. In all animal experiments, 4 mice were used in each test and control group, processed according to specific methods described below. Images shown are representative of each group.

### *In vitro* Treatment of Mouse Brain Tissue with Targeted Liposomes

Brain tissue slices from APP/PSEN1 transgenic mice were incubated in a solution of targeted liposomes (1 µM, in 10 mM Tris-HCl, pH 7.4), at room temperature for 1 h. The sections were then washed extensively with Tris-HCl to remove unbound liposomes, mounted with Vectashield media and viewed under a confocal microscope (Olympus IX61 DSU). Images were processed with Neurolucida (Microbrightfield). Brain tissue sections from control animals and young mice with no amyloid plaques, were also processed following similar procedures and stained as described above, to rule out non-specific binding.

### Intravenous Delivery of Targeted Liposomes to Cortical and Hippocampal Plaques in APP/PSEN1 Transgenic Mice

APP/PSEN1 transgenic mice were anesthetized by isofluorane inhalation. Targeted liposomes were then injected via the tail vein, at a lipid dose of 133 µmol/kg. The animals were euthanized 72 h post-injection and the brain excised. The brain was fixed in 10% formalin and stored in 30% sucrose prior to sectioning. A cryotome (Leica) was used to obtain 30 µm coronal sections of the whole brain. The tissue sections were transferred into phosphate-buffered saline, pH 7, and stored at 4°C. The sections were then mounted with Vectashield media and viewed under an Olympus IX61 DSU microscope using a DAPI filter set (Brightline DAPI-506B-OMF, Semrock, Rochester, NY); Similar to XO4, the labeled liposomes exhibit a peak fluorescence at 430 nm.

### *In vitro* Immunofluorescence Study of Treated Mouse Brain Sections to Confirm Particle Localization on Aβ Plaques

Brain sections from treated mice were transferred to wells and washed twice with Tris-buffered saline with 0.2% tween (PBST). The sections were then incubated with 5% normal donkey serum (NDS) in PBST for 1 h. This was followed by separate incubations with the primary antibody against amyloid-β (anti-mouse amyloid-β antibodies DE2B4 and 4G8) in 3% NDS at 4°C overnight. The negative controls were not incubated with the primary antibody. Sections were then brought to room temperature and washed three times (5 min each) with PBST and incubated with the FITC-labeled secondary anti-mouse IgG (for DE2B4) and cy5-tagged Dylight649 (for 4G8) for 1 h at room temperature. Finally, the sections were washed 4 times, 5 min each with TBST. After the final wash the sections were mounted on glass slides in Vectasheild (Vector Laboratories) and stored at 4°C. Images shown are representatives from 5 slices collected from 4 mice in each group. All images were used for quantitative analysis. In each image, the punctate XO4 signal was used to tessellate the field around each focal plaque (shown by the thin red lines in each image) and create contiguous subdomains within which the co-localization could be assessed. Each subdomain was masked into two regions: the “nuclear” region corresponding to the pixels of the XO4 signal, and the “protein” region, corresponding to all other pixels in the subdomain. Co-localization was then quantified by the Pearson’s Correlation coefficient (PCC) of nuclear intensity versus protein intensity over nuclear mask (PCC NiPiNm). All calculations were performed using Cytseer software (Vala Sciences). The graphs (**M** and **N**) to the right of the images show the PCC values for each treatment. Means and standard deviations were calculated over 156 focal plaques for the DE2-B4 antibody and 40 focal plaques for the 4G8 antibody.

### *Ex vivo* Liposome Co-localization Study

Rhodamine was encapsulated into XO4-targeted liposomes by the passive loading method, at a concentration of 10 mM as described in the liposome preparation section above. 72 h after intravenous injection in anesthetized mice, the animals were sacrificed, the brains fixed in 4% paraformaldehyde for 48 h at 4°C, transferred to 30% sucrose in 1X-PBS at 4°C until the tissue sank (usually about 72 h). The tissue was then embedded in OCT compound (Sakura Finetek, Torrance CA) and stored at −80°C until serial sectioning. Sections were cut at 30 µm thickness, and stored at 4°C in PBS until mounted. The sections were then mounted with Vectashield media and viewed under an Olympus IX61 DSU microscope Imaging was conducted in two channels using a 20X objective. Liposomal MeXO4 was imaged using a Brightline DAPI-506B-OMF filter set (Semrock; Rochester, NY); Rhodamine was imaged using a Brightline TRITC-A-OMF filter set (Semrock; Rochester, NY). Images were processed with Neurolucida (Microbrightfield).

## Supporting Information

Information S1 **^1^H NMR and ^13^C NMR spectra of all new compounds; ^1^H NMR and MALDI mass analysis of conjugate; TEM image of liposomes and liposome stability test data.**
(PDF)
