# Supplementary material for: Intravenous Delivery of Targeted Liposomes to Amyloid-β Pathology in APP/PSEN1 Transgenic Mice
Source: PLoS One. 2012 Oct 31;7(10):e48515. doi: 10.1371/journal.pone.0048515 (PMC3485335; doi:10.1371/journal.pone.0048515)
Supplement: Information S1 — 1H NMR and 13C NMR spectra of all new compounds; 1H NMR and MALDI mass analysis of conjugate; TEM image of liposomes and liposome stability test data. (PDF) [file pone.0048515.s001.pdf]

Intravenous Delivery of Targeted Liposomes to Amyloid-β Plaques in APP/PSEN1 Transgenic Mice

Eric A. Tanifum,[1‡] Indrani Dasgupta,[2†] Mayank Srivastava,[1‡] Rohan C. Bhavane,[1‡] Li Sun,[3] John Berridge,[3] Hoda Pourgarzham, [3] Rashmi Kamath,[1] Gabriela Espinosa,[1] Stephen C. Cook,[4] Ananth Annapragada,[1‡*] Jason L. Eriksen[2*]

[1]Pediatric Radiology, Texas Children's Hospital, Houston, TX 77030
[2]School of Biomedical Informatics, The University of Texas Health Science Center at Houston, Houston, TX 77030
[3]Department of Pharmacology and Pharmaceutical Sciences, University of Houston, Houston, TX 77204
[4]Alzeca Biosciences, Houston, TX 770098
[†]Current address: Department of Anatomy & Neurobiology, University of Maryland School of Medicine, Baltimore, MD 21201
[‡]Formerly of School of Biomedical Informatics, The University of Texas Health Science Center at Houston, Houston, TX 77030
[*]Corresponding authors: avannapr@texaschildrens.org and jeriksen@central.uh.edu

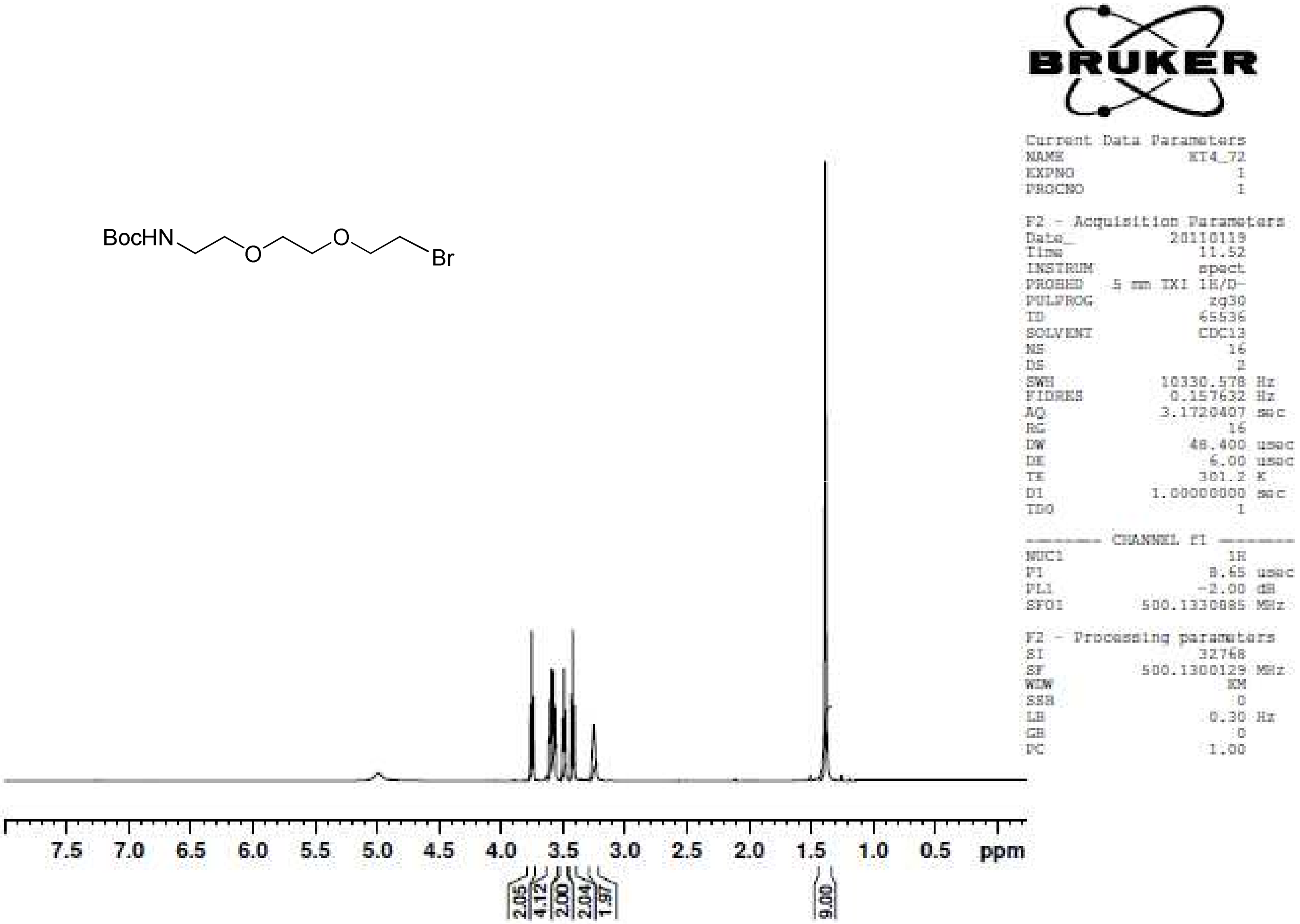

Figure S1. ^{1}H NMR of compound **3**

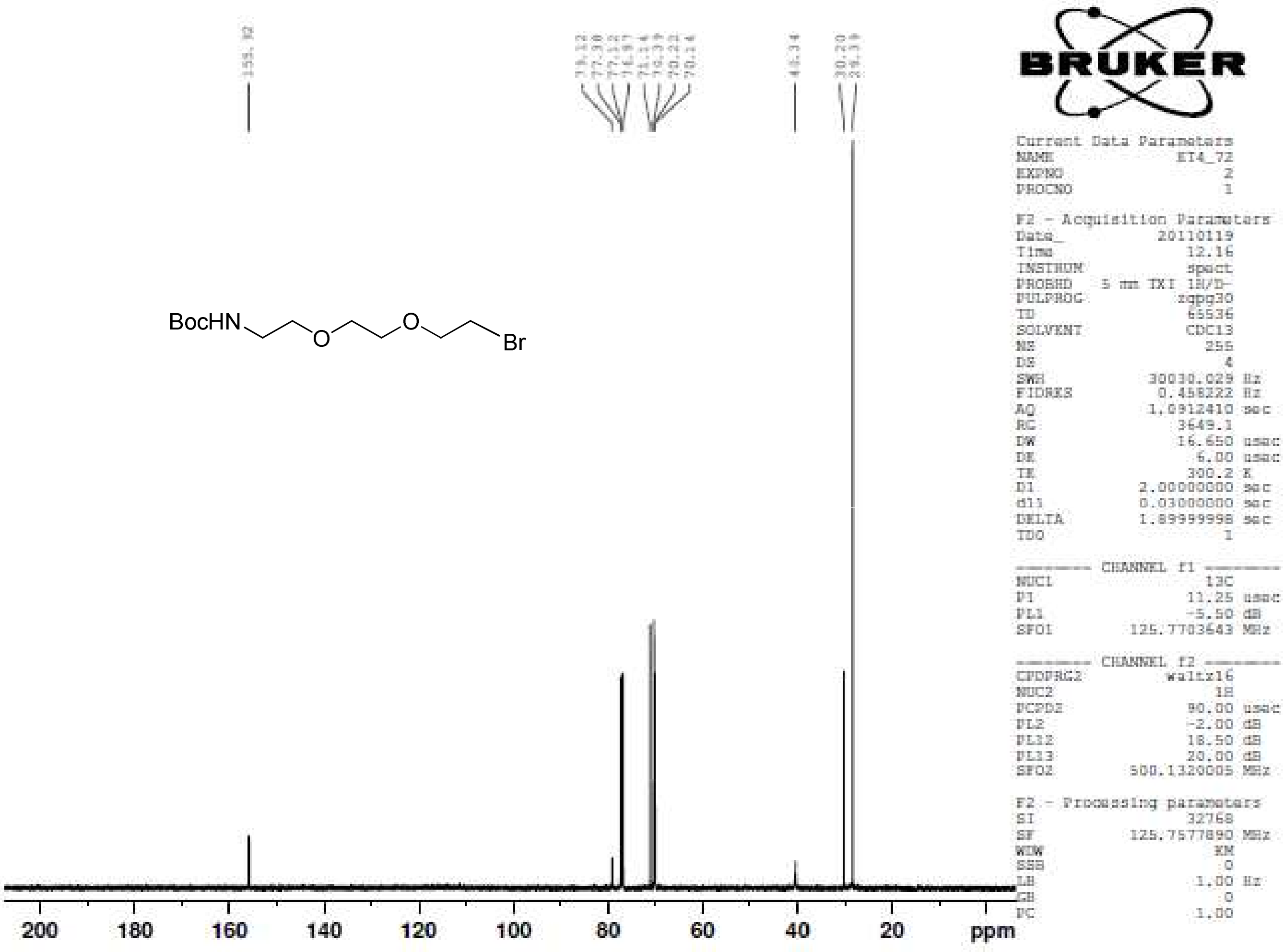

Figure S2. ^{13}C NMR of compound **3**

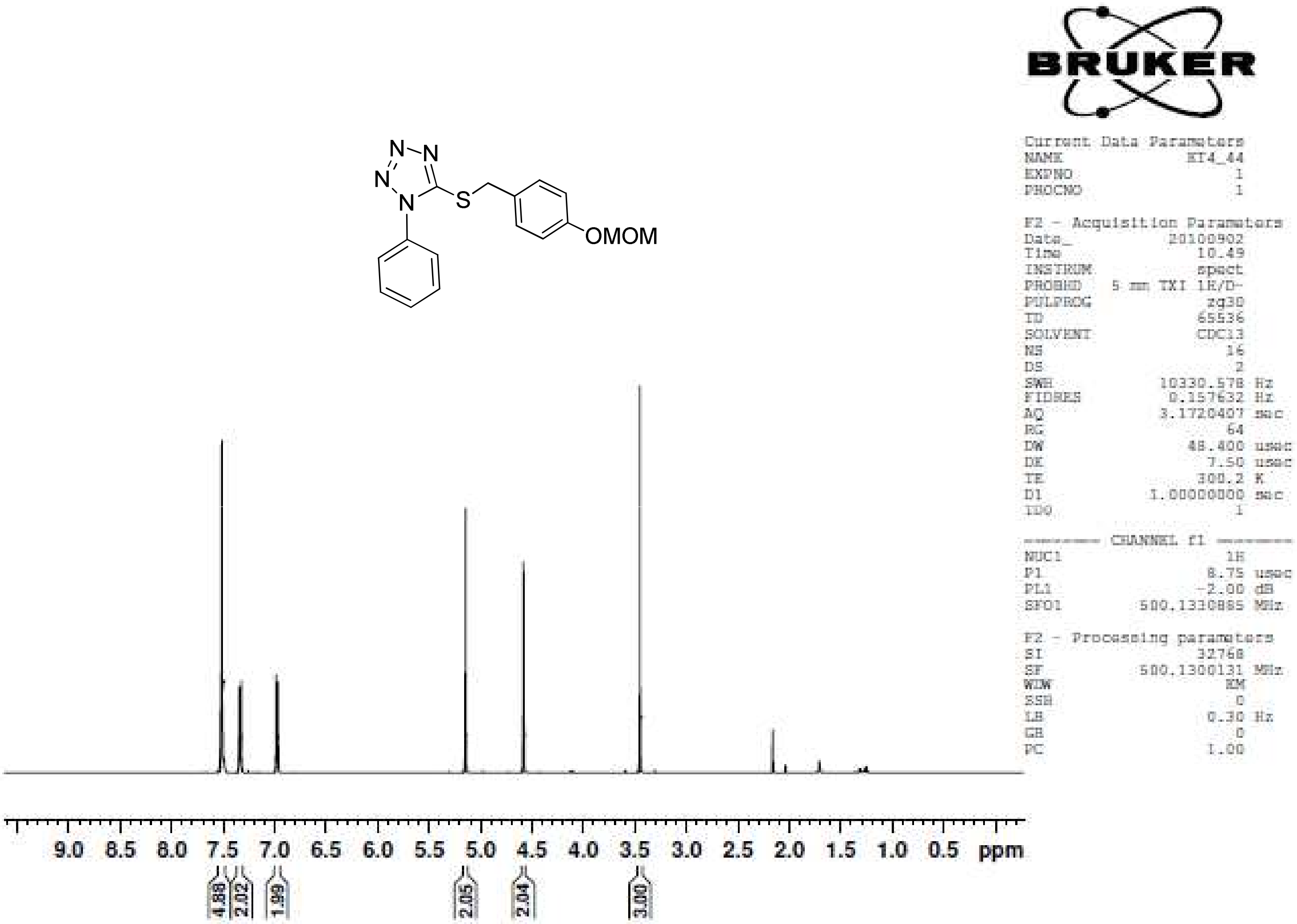

Figure S3. ^{1}H NMR of compound **6**

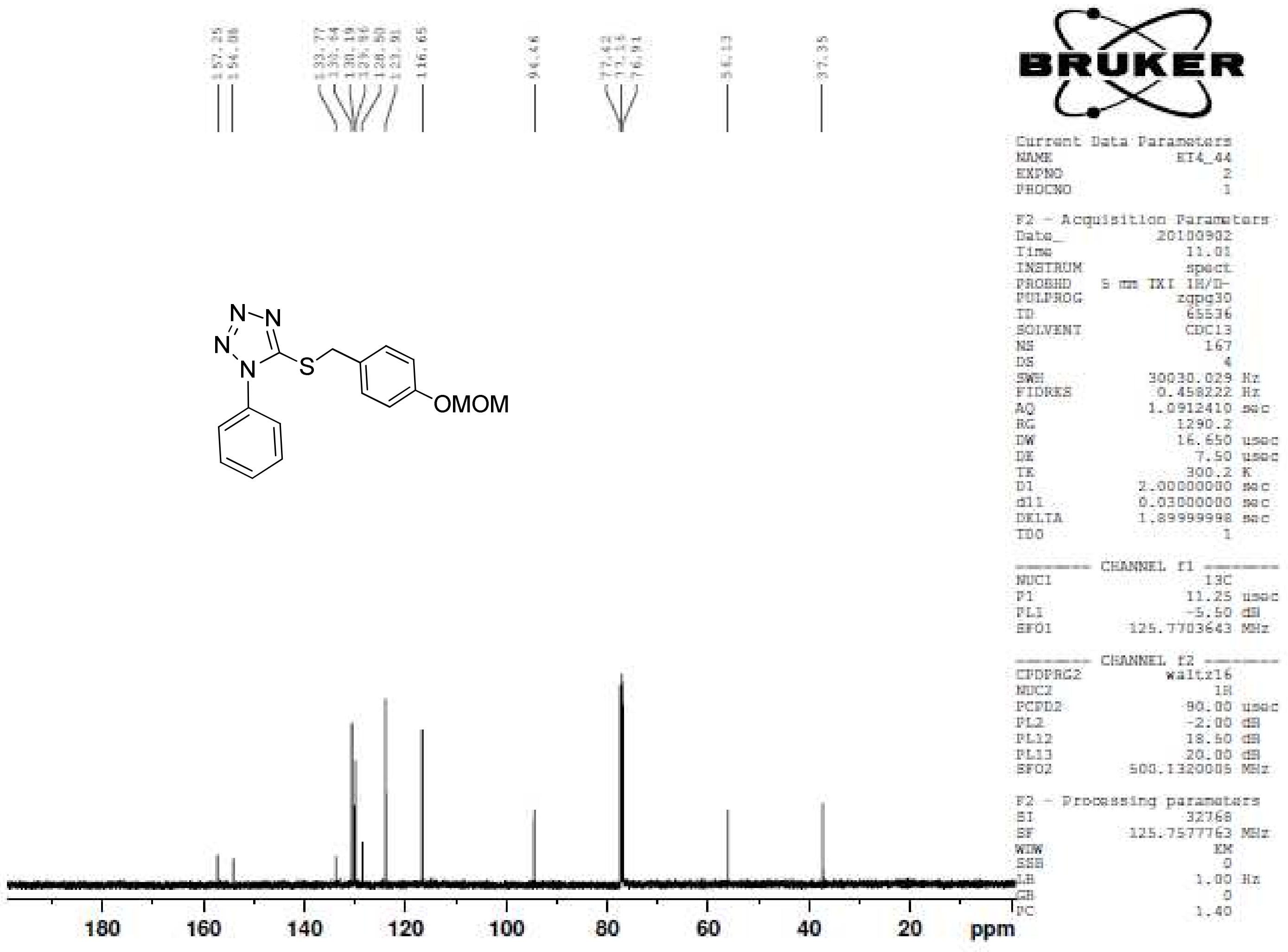

Figure S4. ^{13}C NMR of compound **6**

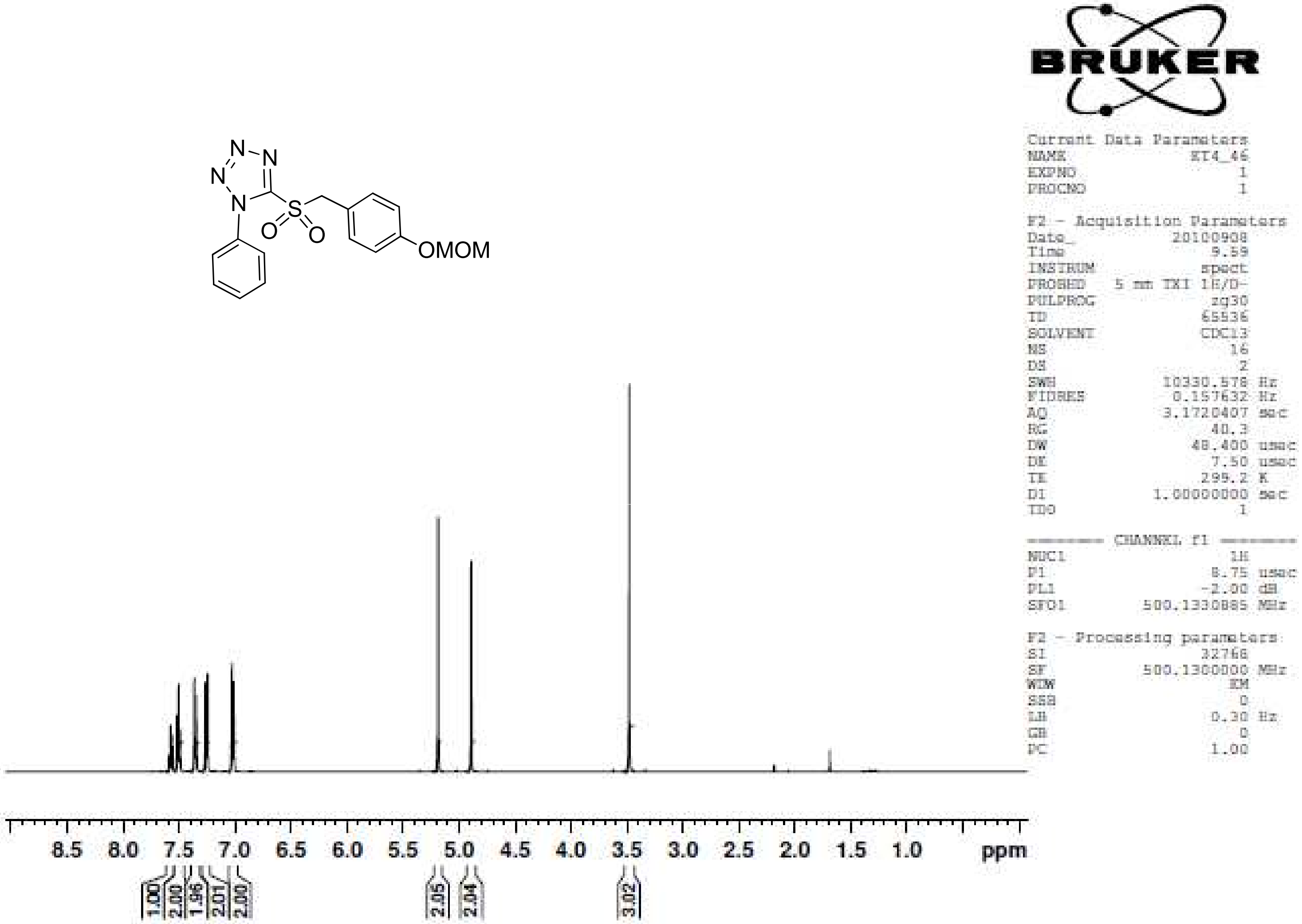

Figure S5. ^{1}H NMR of compound **7**

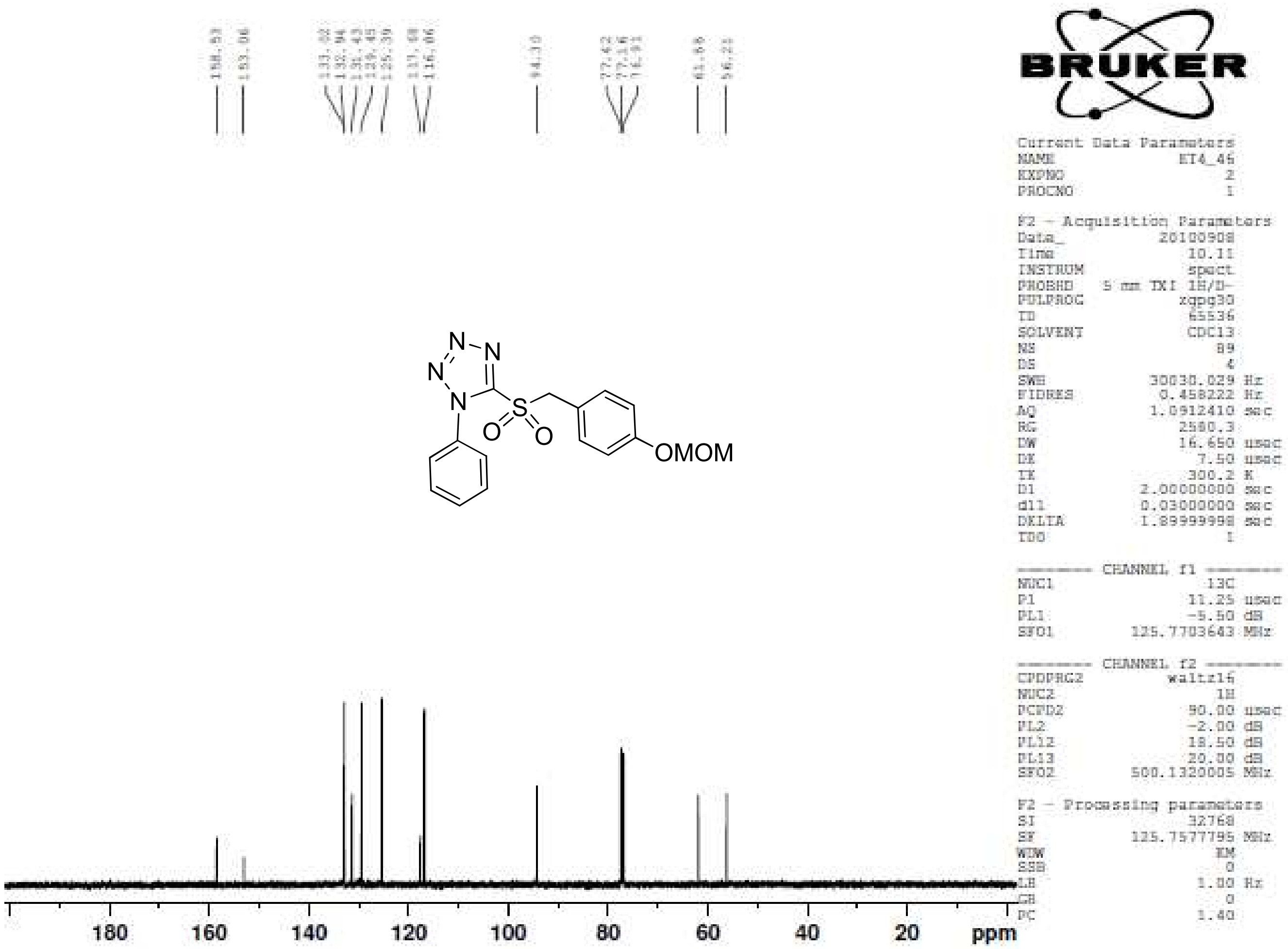

Figure S6. ^{13}C NMR of compound **7**

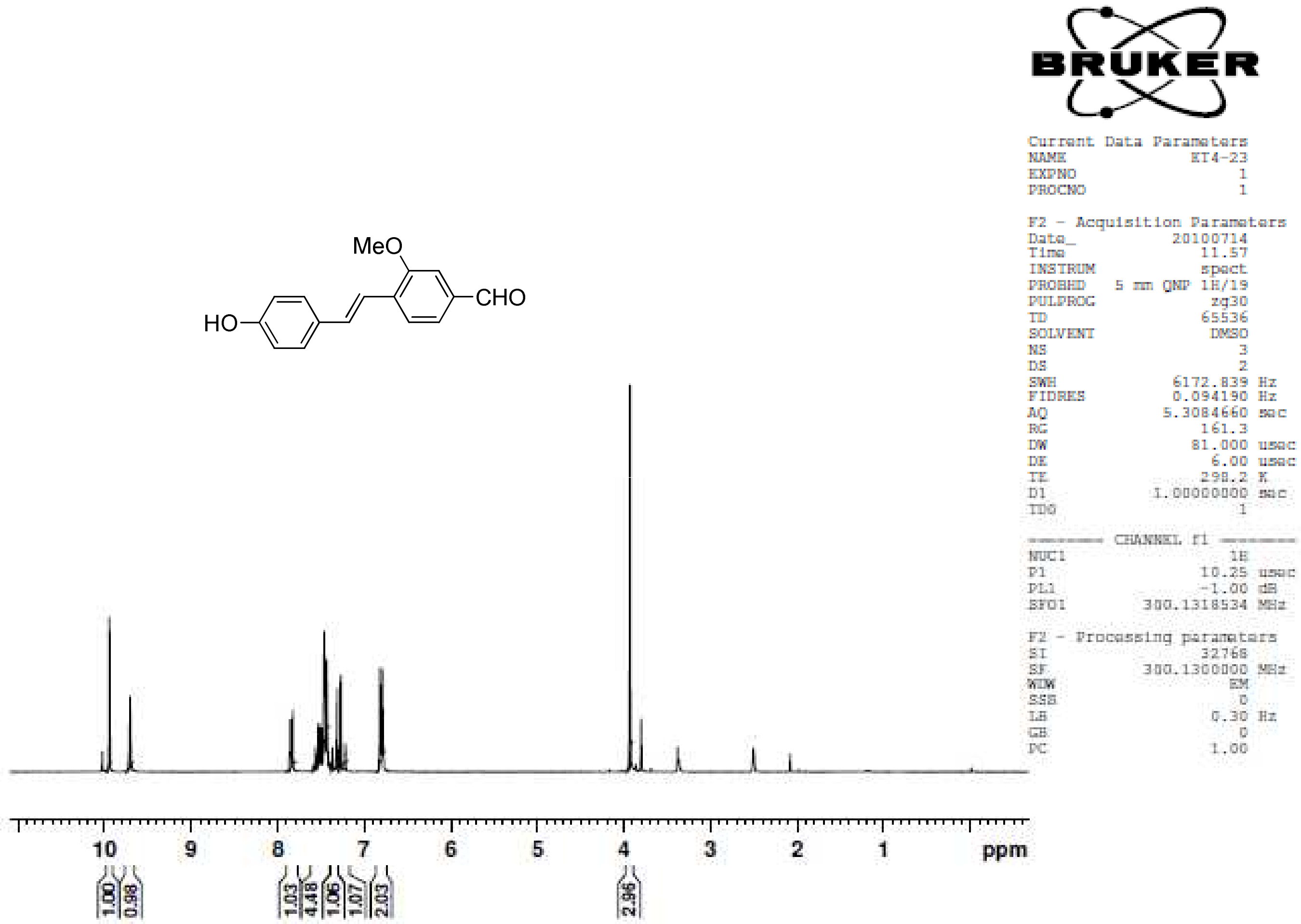

Figure S7. ^{1}H NMR of compound **10**

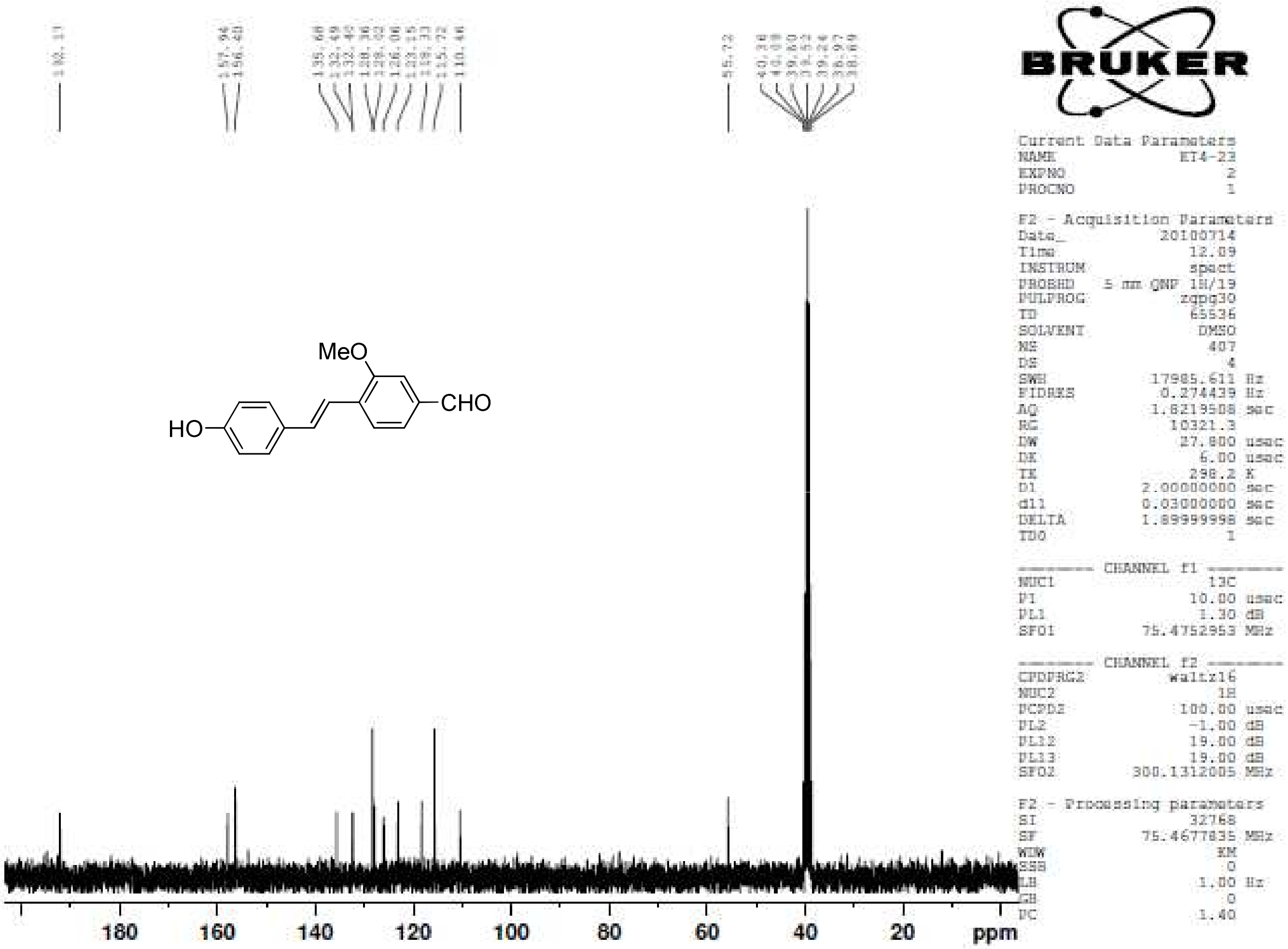

Figure S8. ^{13}C NMR of compound **10**

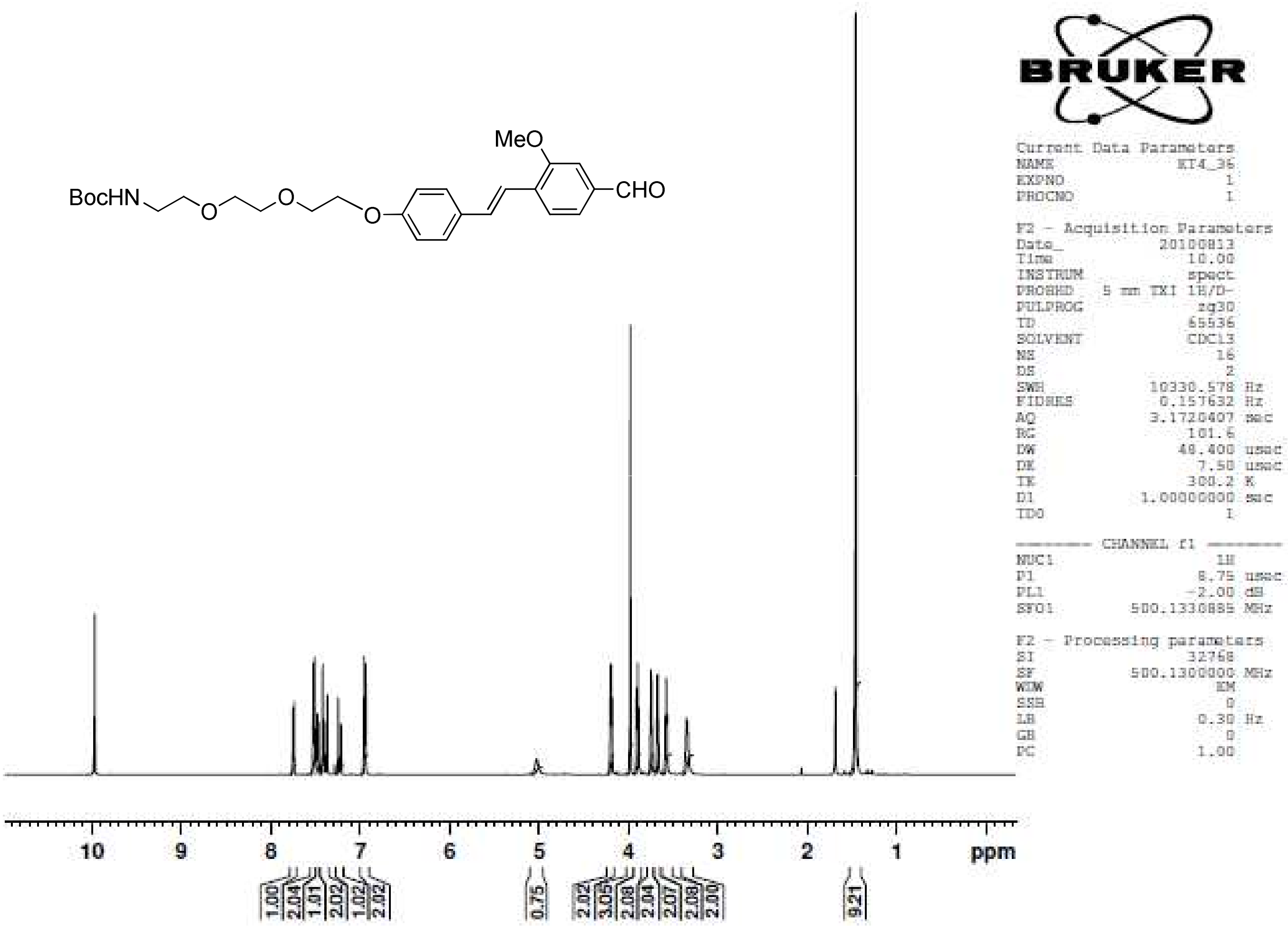

Figure S9. ^{1}H NMR of compound **11**

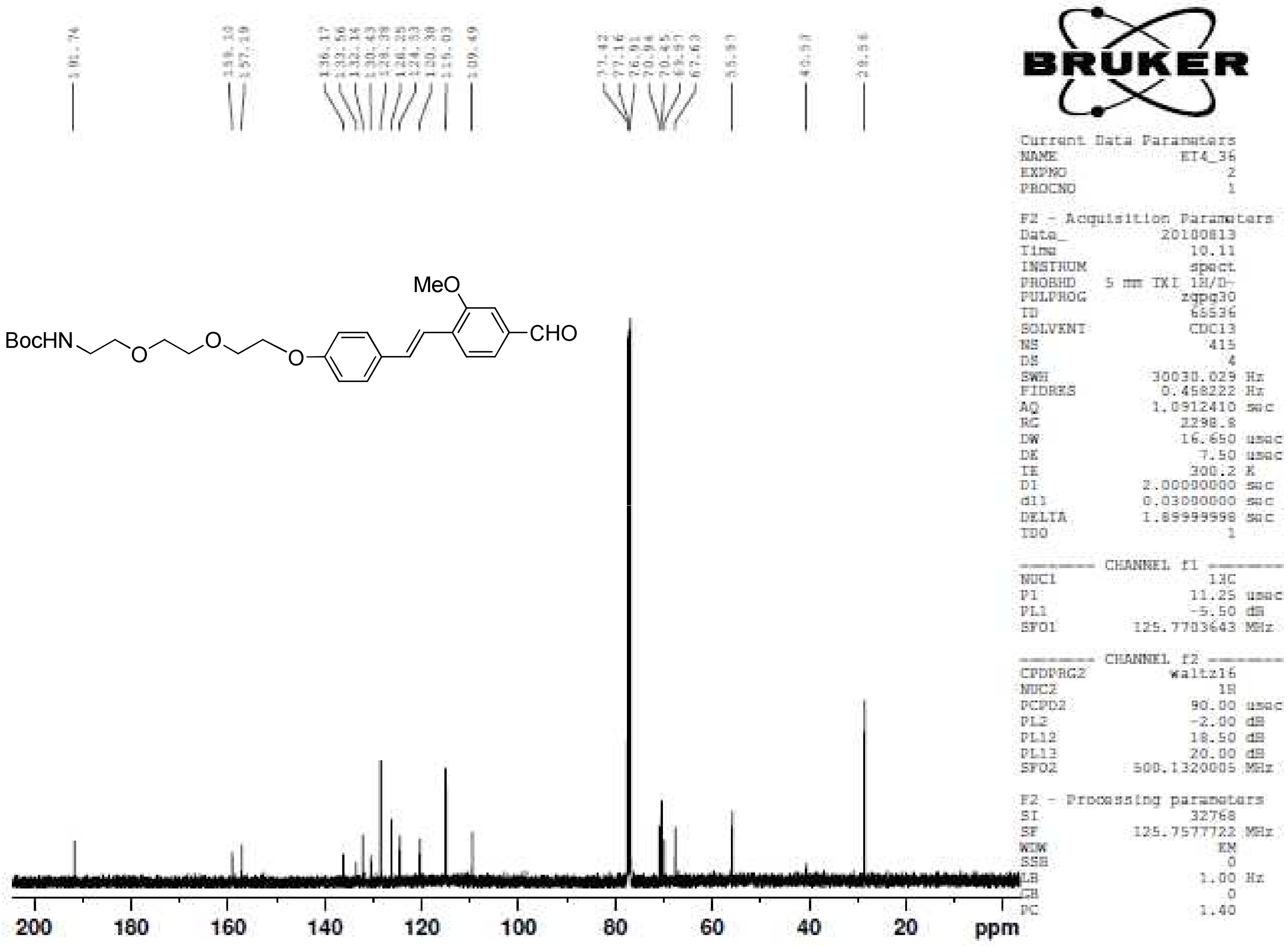

Figure S10. ^{13}C NMR of compound **11**

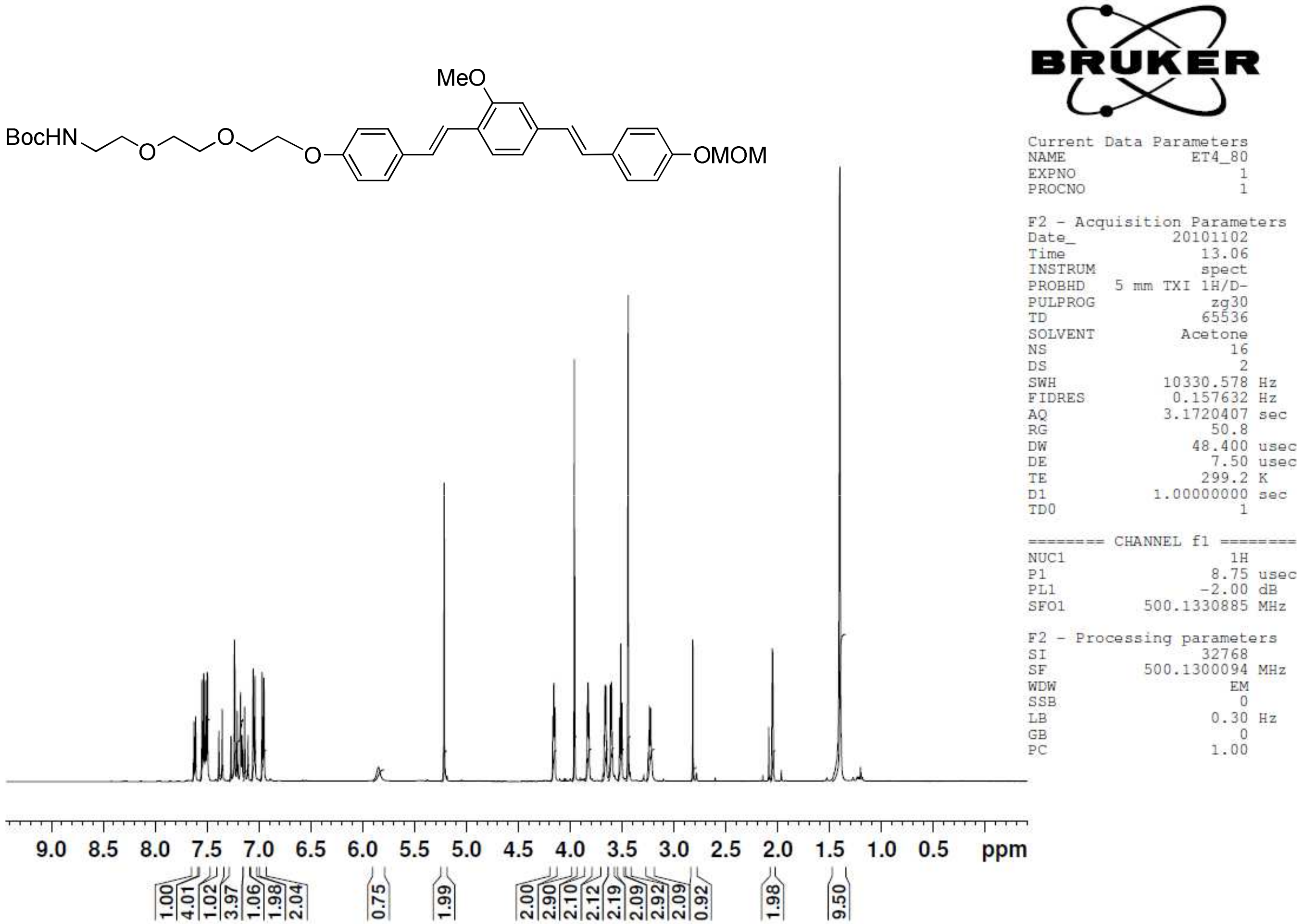

Figure S11. ^{1}H NMR of compound **12**

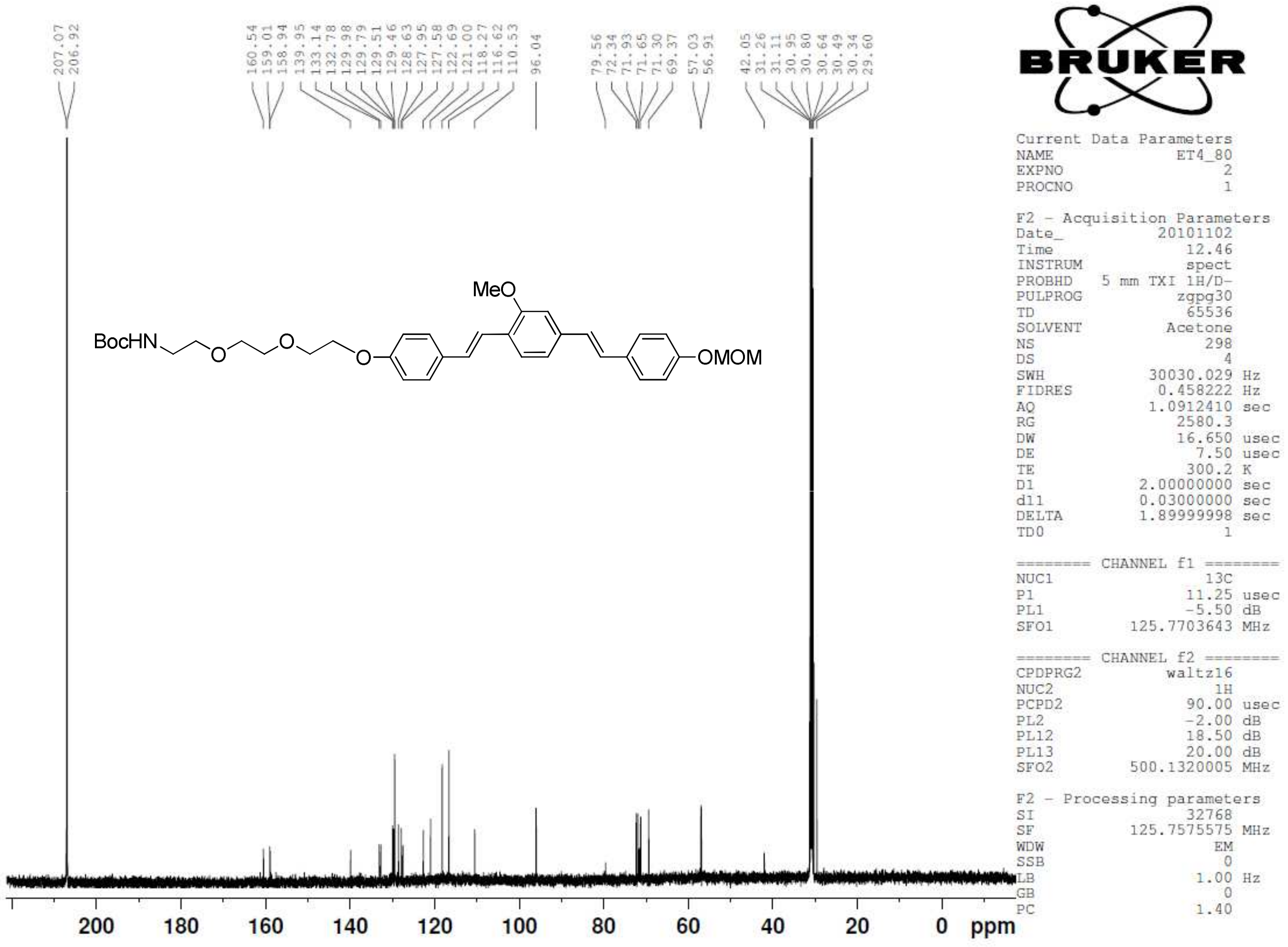

Figure S12. ^{13}C NMR of compound **12**

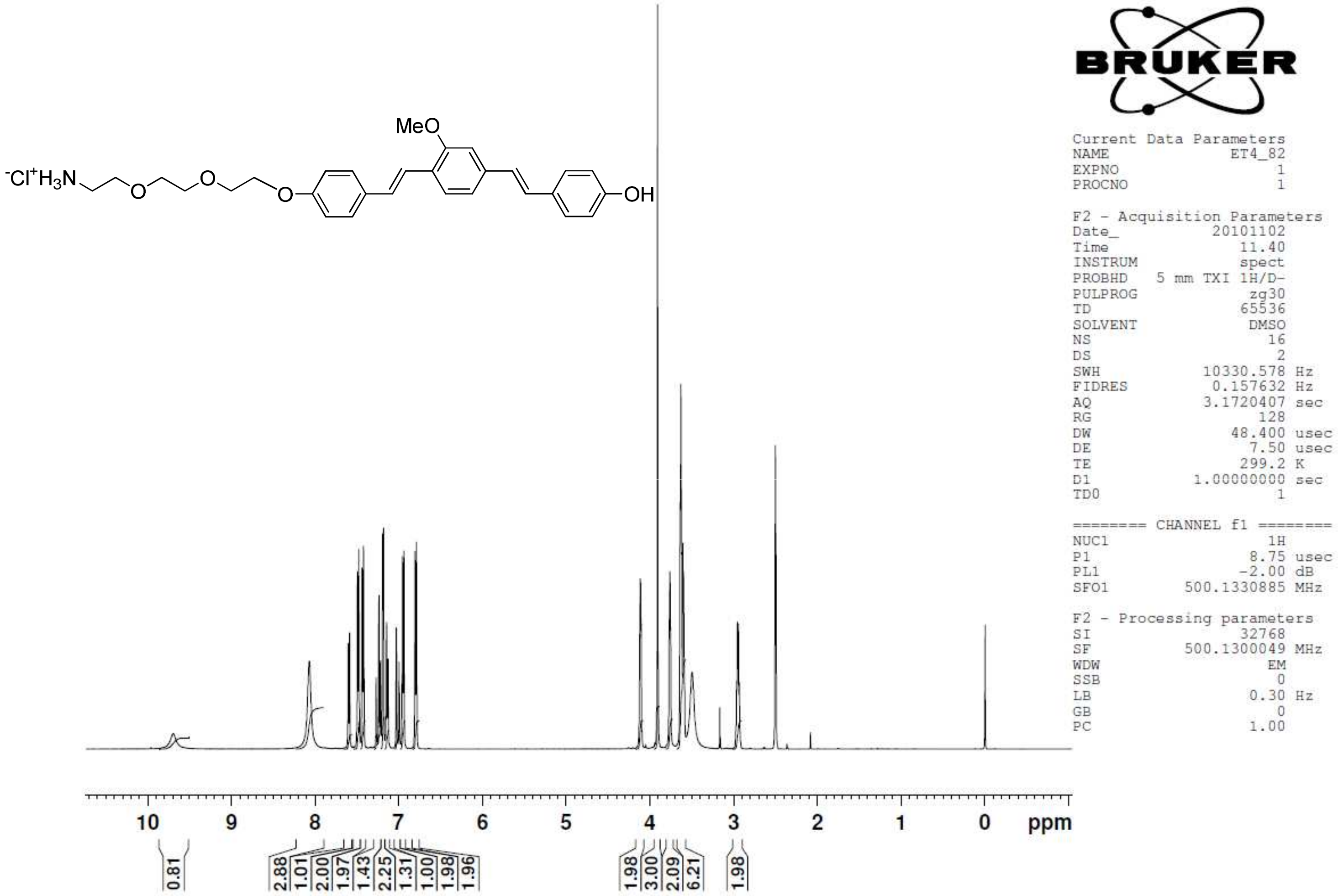

Figure S13. ^{1}H NMR of compound **13**

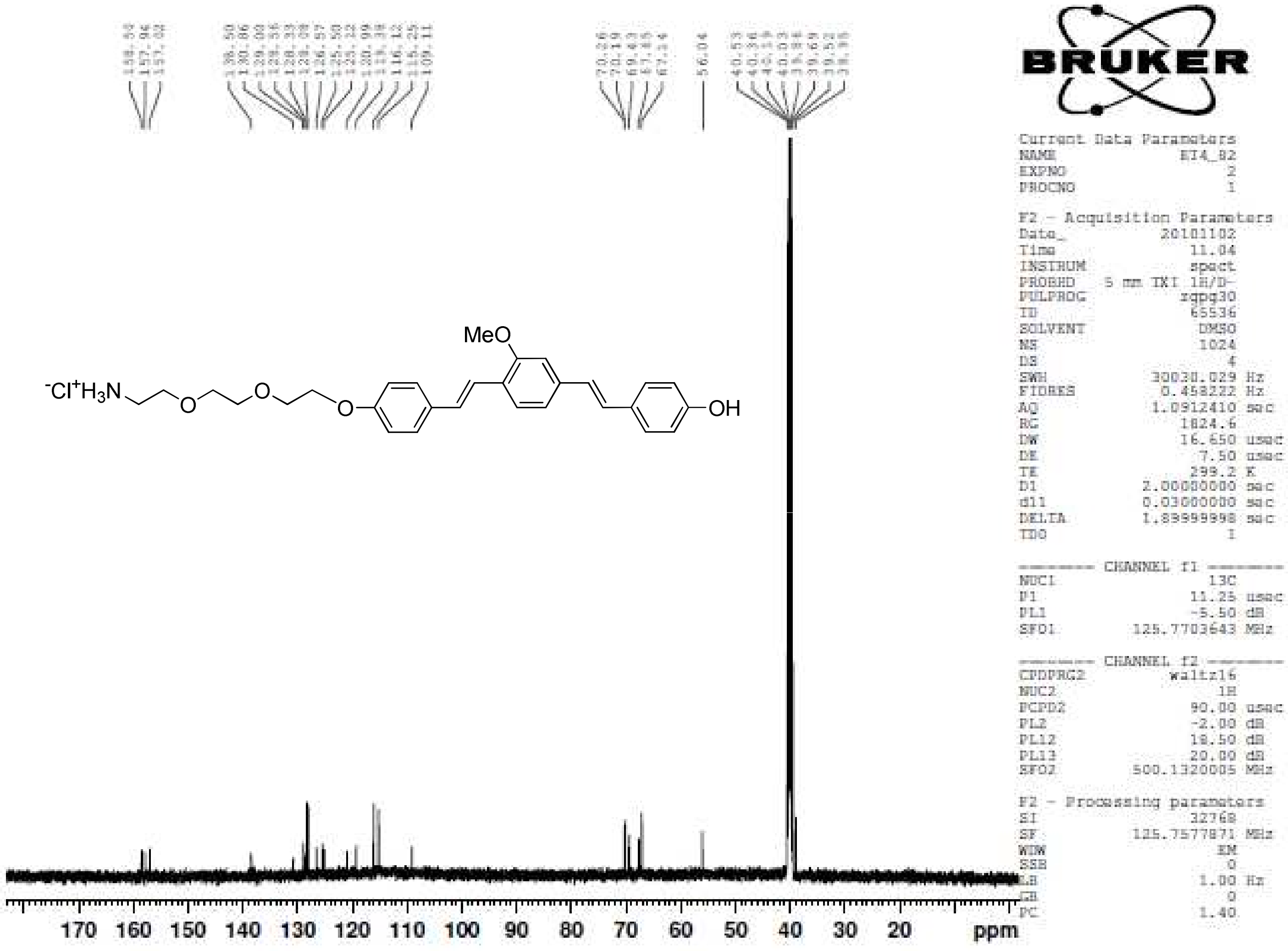

Figure S14. ^{13}C NMR of compound **13**

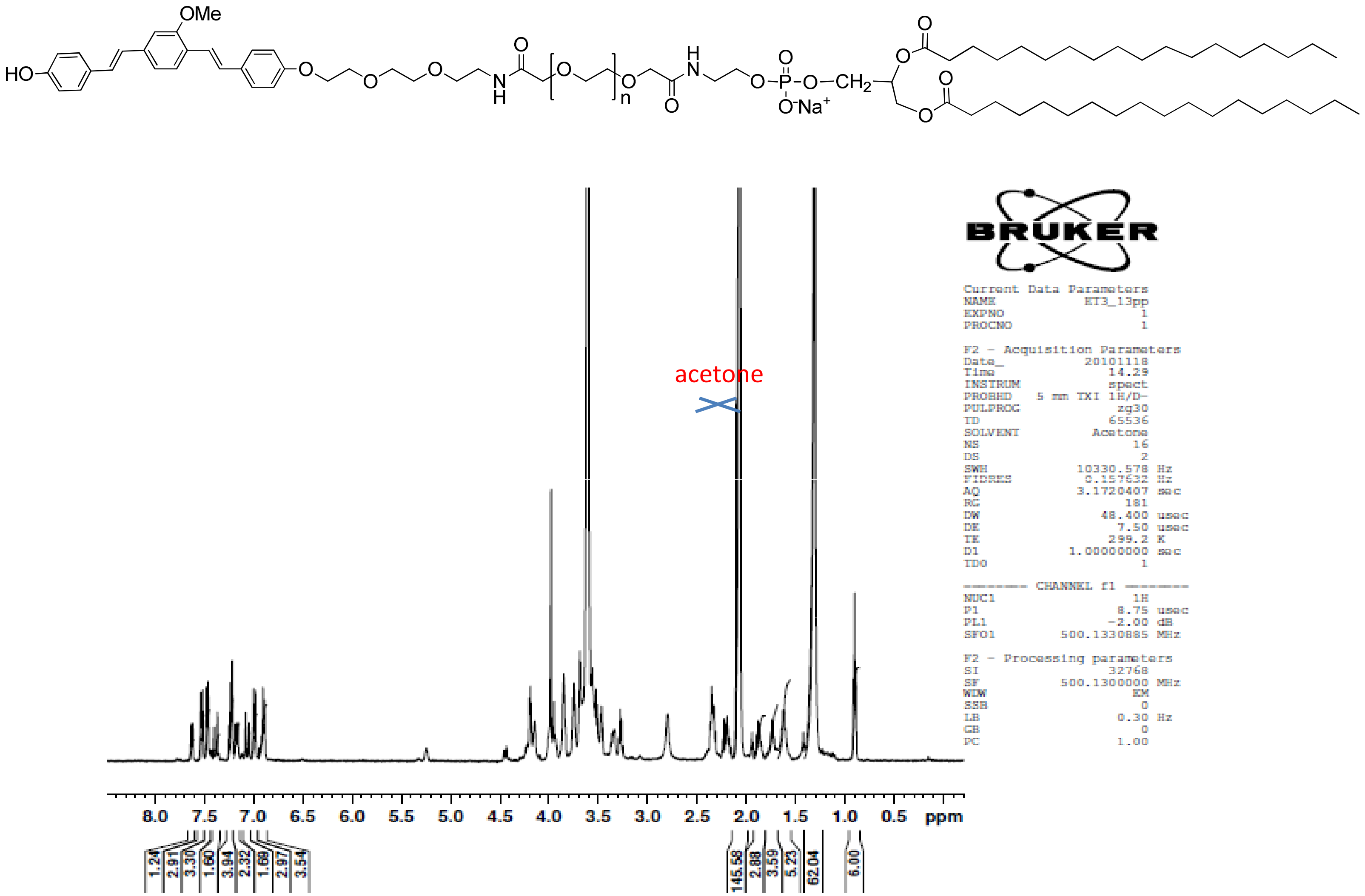

Figure S15. ^{1}H NMR of DSPE-PEG$_{3400}$-MeXO4 (**1**)

D:\Data\2010\Dec 2010\M120110\16096 ET3-13\0_O10\1

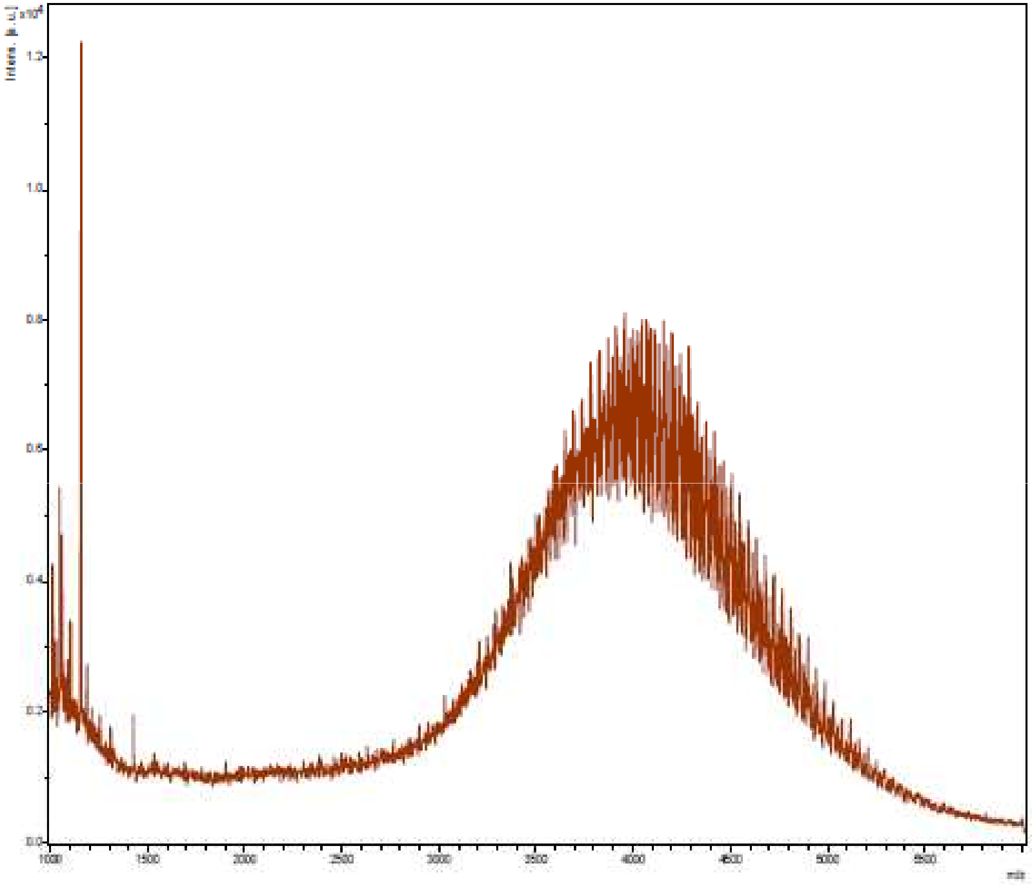

m/z

m/z	S/N	Quality Fac.	Res.	Intens.	Area
3459.624	2.4		17461	4618.00	464
3474.843	2.4		7609	4681.00	715
3503.765	2.4		9966	4982.00	1043
3517.816	2.4		32478	5027.00	370
3546.625	2.3		5609	5130.00	834
3562.383	2.5		17571	5395.00	441
3580.133	1.7		17309	4960.00	232
3591.427	2.7		7538	5649.00	1096
3605.687	2.7		6039	5651.00	1155
3623.712	2.3		16259	5586.00	464
3635.828	2.0		8732	5387.00	499
3649.761	3.1		9088	6185.00	1391
4069.004	5.2		5219	8000.00	2772
4089.414	5.0		7688	7881.00	2284
4112.390	4.6		8824	7519.00	2225
4132.763	4.8		21505	7621.00	1178
4156.905	5.4		6245	7910.00	2887
4177.586	4.1		9262	7016.00	1751
4200.754	5.5		40615	7810.00	1318
4221.881	4.8		44297	7293.00	831
4245.203	5.4		9678	7486.00	2142
4265.398	4.4		7817	6826.00	1334
5032.262	2.4		14687	2167.00	495
5055.144	1.6		73822	1800.00	109
5076.077	2.0		43608	1886.00	297
5097.702	1.3		16786	1537.00	153
5120.206	1.8		19808	1699.00	312
5143.234	1.5		25748	1503.00	209
5163.639	1.2		10727	1353.00	289
5187.326	1.2		12082	1309.00	182
5207.960	1.6		10537	1402.00	277

12/1/2010 6:51:30 AM

Figure S16. MALDI spectrum of DSPE-PEG$_{3400}$-MeXO4 (**1**)

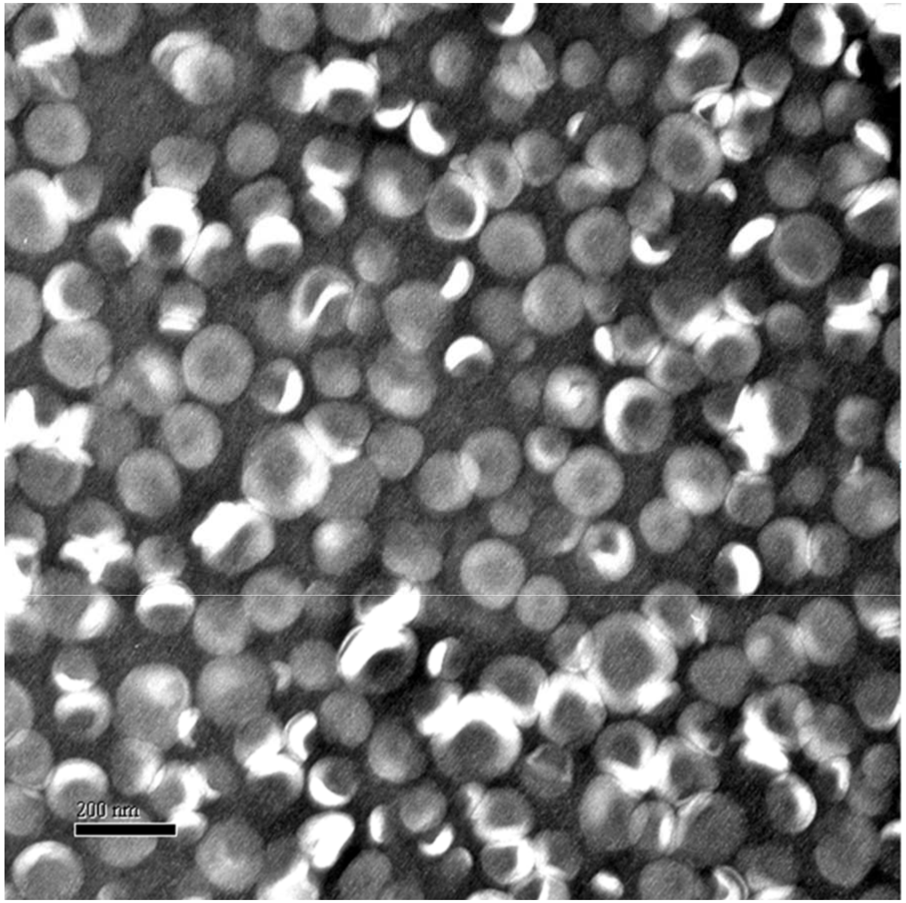

Figure S17. Negative Stain TEM of targeted liposomes. Grid with sample was incubated with Uranyl Acetate for 2 minutes 3 times, followed by imaging at 80kV. Exposure time was 0.9 seconds, effective magnification 12000x.

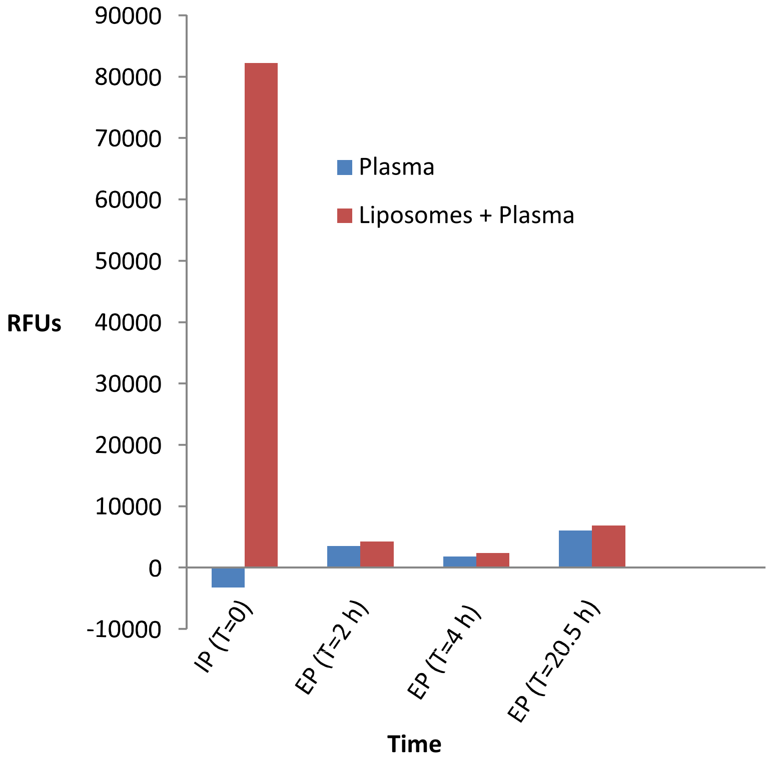

Figure S18. Liposome stability-Plasma leak test results: To evaluate the stability of the particles in plasma, 300 μL of the targeted liposomal preparation was added to 1 mL of bovine plasma in histidine/saline buffer (10 mM, pH 7.4) and incubated at 37 ℃ for 90 min. This mixture (internal phase, IP) was placed in a dialysis cassette (100,000 MWCU) and dialyzed against 100 mL of buffer (external phase, EP). 1 mL plasma was subjected to the same protocol to serve as control. At the start of the dialysis 3 μL each of the internal phase of both test and plasma (control) were diluted to 300 μL in ethanol and the fluorescence of the resulting mixture measured at 450 nm (IP, T=0). The fluorescence of 300 μL samples taken from the external phase of both test and control were measured as 2 h, 4 h, and 20.5 h time points (EP, T=2 h; EP, T=4 h; EP, T=20.5 h) respectively. As can be seen from the data above, the integrity of the liposomes remains intact during the course of the experiment as disintegration of the particles would have resulted in a spike in fluorescence in the external phase.
